# The moving-beam diffraction geometry: the DIAD application of a diffraction scanning probe

**DOI:** 10.1107/S1600576725009811

**Published:** 2026-02-01

**Authors:** Alberto Leonardi, Andrew James, Christina Reinhard, Michael Drakopoulos, Ben Williams, Hans Dehyle, Jacob Filik, Liam Perera, Sharif Ahmed

**Affiliations:** a Physical Sciences Diamond Light Source Diamond House – Harwell Science and Innovation Campus Didcot OxfordshireOX11 0DE United Kingdom; b The University of Manchester at Harwell Harwell Science and Innovation Campus Didcot OxfordshireOX11 0DE United Kingdom; c Faculty of Science and Engineering The University of Manchester Oxford Road ManchesterM13 9PL United Kingdom; d National Synchrotron Light Source II Brookhaven National Laboratory Bldg 741 PO Box 5000 Upton NY11973-5000 USA; e Biomaterials Science Center (BMC) University of Basel Hegenheimermattweg 167C Basel Basel-StadtCH-4123 Switzerland; Australian Synchrotron, ANSTO, Australia

**Keywords:** moving-beam diffraction geometry, diffraction scanning probes, area-detector diffraction-geometry calibration, DIAD beamline, nearest-neighbor geometry calibration

## Abstract

Understanding the interactions between microstructure, strain, phase and material behavior is crucial in scientific fields such as energy storage, carbon sequestration and biomedical engineering. However, quantifying these correlations is challenging, as it requires the use of multiple instruments and techniques, often separated by space and time. The Dual Imaging and Diffraction (DIAD) beamline at Diamond Light Source is designed to address this challenge. DIAD allows its users to visualize internal structures (in two and three dimensions), identify compositional/phase changes and measure strain. It enables *in situ* and *operando* experiments that require spatially correlated information. DIAD provides two independent beams combined at one sample position, allowing ‘quasi-simultaneous’ X-ray computed tomography and X-ray powder diffraction. A unique functionality of the DIAD configuration is the ability to perform ‘image-guided diffraction’, where the micrometre-sized diffraction beam is scanned over the complete area of the imaging field of view without moving the specimen. This moving-beam diffraction geometry enables the study of fast-evolving and motion-susceptible processes and samples. Here, we discuss the novel moving-beam diffraction geometry, presenting the latest findings on the reliability of both the geometry calibration and the data-reduction routines used. We provide a comprehensive quantitative assessment of the moving-beam diffraction geometry implemented at the DIAD beamline, which will serve as a reference for beamline users. Our measurements confirm that diffraction is most sensitive to the moving-beam geometry for the conventional transmission geometry of the detector. The observed data confirm that the motion of the Kirkpatrick–Baez mirror coupled with a fixed-aperture slit results in a rigid translation of the beam probe, without affecting the angle of the incident-beam path to the sample. Our measurements demonstrate that a nearest-neighbor calibration can achieve the same accuracy as a self-calibrated geometry when the distance between the calibrated and probed sample regions is smaller than or equal to the beam spot size. The absolute error of the moving-beam diffraction geometry at DIAD with typical calibration setup remains below 0.01%, which is the accuracy we observe for the beamline with stable beam operation.

## Abstract 2

We introduce the moving-beam diffraction geometry as implemented at the Dual Imaging and Diffraction beamline at Diamond Light Source, and provide a quantitative assessment of the precision offered by this geometry and the nearest-neighbor calibration method.

## 1. Introduction

**Figure 1:**
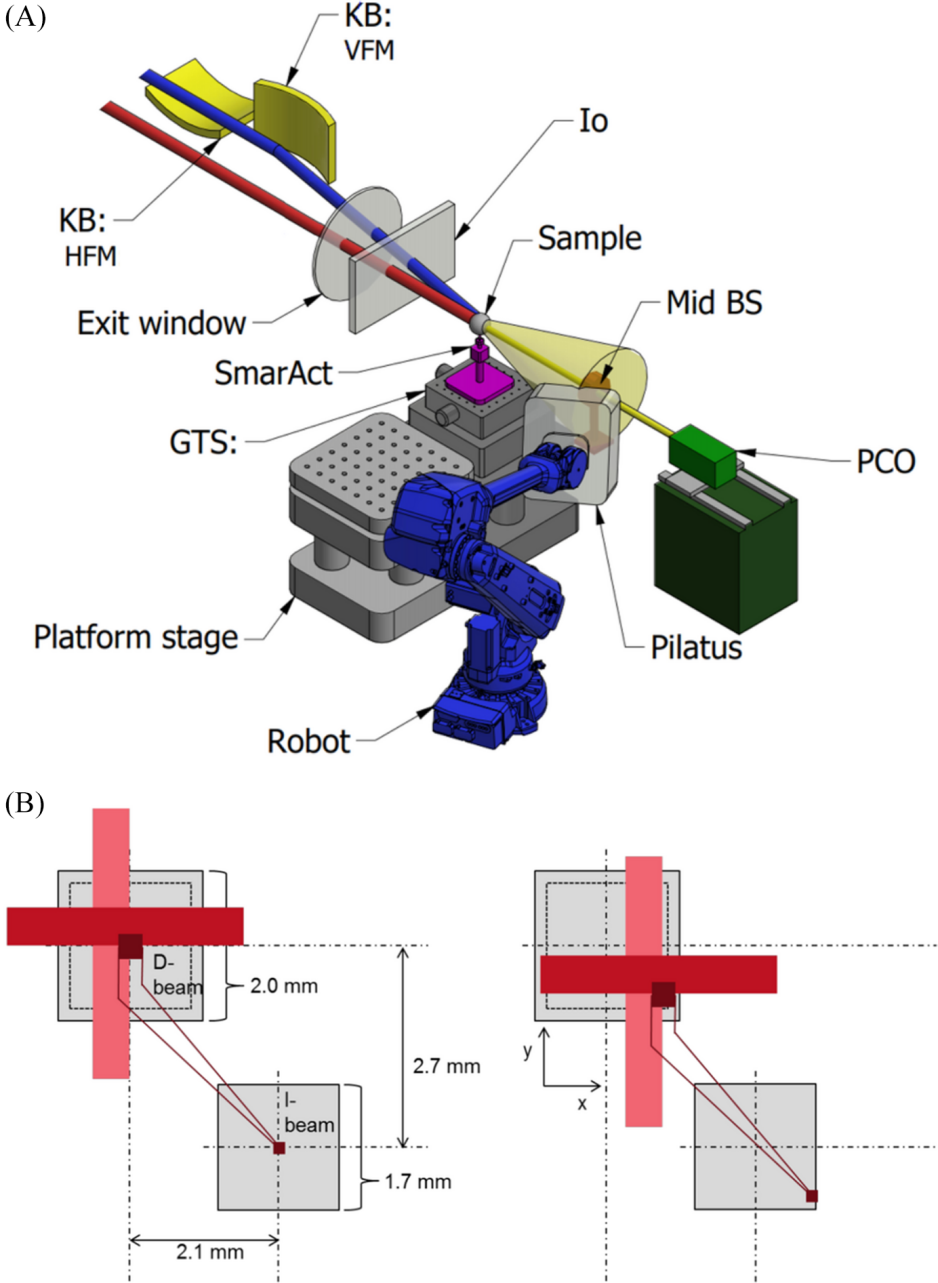
Schematic drawings of the moving-beam scanning that is used on DIAD. (*A*) Spatial configuration of the optical elements within the experimental hatch, showing the imaging (red) and diffraction (blue) beam paths. The KB focusing mirror refocuses the diffraction beam onto the sample position, which is spatially registered with the imaging FOV. HFM: horizontal focusing mirror. VFM: vertical focusing mirror. Mid BS is the beamstop used to stop the direct beam from hitting the diffraction detector. (*B*) A set of fixed-aperture slits, mechanically coupled to the KB mirrors, selects a sub-region of the diffraction beam. The horizontal and vertical KB mirrors, equipped with fixed-aperture plates, focus the dark red portion of the diffraction beam onto the sample. Due to the angular offset between the diffraction and imaging beam paths, the diffraction beam is focused across a FOV (∼1.7 mm as per DIAD design) located ∼2 mm from the projected position of the KB slits.

Spatially correlated imaging and scattering information enables the study of complex systems across different spatial and temporal scales, such as energy-storage materials, soil mechanics, bio-medical materials, and heritage or space-mission samples (Besnard *et al.*, 2023 ▸, 2024 ▸; Le Houx *et al.*, 2023 ▸; Reed *et al.*, 2024 ▸; Vashishtha *et al.*, 2024 ▸). Extending from the opportunity of *ex temporal* observations available at other instruments (Drakopoulos *et al.*, 2015 ▸; King *et al.*, 2016 ▸), the Dual Imaging and Diffraction (DIAD) beamline at Diamond Light Source provides inherent space and time correlation between structural and morphological information (Reinhard *et al.*, 2021 ▸). Two independent synchrotron X-ray beams probe the sample with either imaging or diffraction data collection at a swap frequency rate of 20 Hz, making the corresponding exposure time the time-limiting factor. A rigid set of slits coupled with a Kirkpatrick–Baez (KB) mirror system is mounted on a pair of translation stages to achieve a high-resolution space correlation (Reinhard *et al.*, 2021 ▸). The KB mirror focuses a 300 by 300 µm region of the diffraction beam onto the sample with a spot size ranging from 50 by 50 µm to 15 by 5 µm. Translations of the KB system allow for selective area diffraction mapping by scanning of the focused X-ray microbeam across the imaging volume. While the range of beam translation currently in use is 1.4 by 1.2 mm, which matches the area captured by the PCO camera, an upgrade to an Andor Balor imaging detector is expected to extend the range to 2.0 by 2.0 mm. The live feed from the imaging stream can be used to guide the position of the diffraction beam on the sample (Fig. 1 ▸). Evolving processes can then be probed in space within the limitations of the exposure time for a diffractogram, typically ranging from 2 to 10 s (expected to significantly decrease with the ongoing Diamond ring upgrade), while the KB translation between opposite corners in the field of view (FOV) is achieved in less than 0.5 s.

Scanning the diffraction beam across the sample breaks away from the conventional diffraction scanning approach adopted by other synchrotron beamlines, where the diffraction beam stays stationary and the sample moves (Odstrcil *et al.*, 2019 ▸; Yang *et al.*, 2022 ▸). Moving the X-ray beam instead of the sample eliminates the need to balance high-speed precise movements of the sample stages and the surrounding environment with the requirement to keep the sample undisturbed. This strategy reduces the risk of perturbations such as vibrations, which can, for example, alter the concentration of components in liquids (Noell *et al.*, 2020 ▸). Diffraction and radiography data can be collected without any movement of the sample, enabling the study of processes highly sensitive to vibrations or kinetic effects, such as oxide-reduction reactions in a liquid solution. However, when using a moving beam, the detector pixels measure a different location of the sample’s reciprocal space at each beam position (see Appendix *A*[App appa]). Movement of the diffraction beam also introduces additional complexity into the standard diffraction-data-reduction pipelines. The area-detector data reduction can further magnify instrument aberrations such as peak broadening, peak shifting and peak splitting. Changes in the diffraction geometry affect the instrument’s ability to quantify changes in the diffraction pattern accurately. Aberrations caused by either reflection or transmission diffraction geometries are well known (Borchert, 2014 ▸; Cernik & Bushnell-Wye, 1991 ▸; Cullity, 1956 ▸; Guinier, 1956 ▸; Kriegner *et al.*, 2015 ▸; Warren, 1990 ▸). Common instrument corrections for the analysis of scattering data collected with the Bragg–Brentano or Debye–Scherrer geometries are readily available in most line-profile analysis software tools (Coelho, 2018 ▸; Perl *et al.*, 2012 ▸; Toby & Von Dreele, 2013 ▸). The DIAD moving-beam diffraction geometry is a new way of doing transmission diffraction. Therefore, an additional geometry-calibration step is required for the data-reduction process, which directly affects the resolution of the moving-beam diffraction geometry adopted by the DIAD instrument.

In this work, we present a systematic characterization of the aberrations associated with the moving-beam diffraction geometry implemented at the DIAD beamline. The position and orientation of the diffraction detector relative to a reference X-ray source beam are calibrated by fitting the diffraction pattern from a standard NIST sample. Hence, the diffraction geometry for a KB mirror that has been moved to an arbitrary position (KB position) away from the reference position is either extrapolated on the basis of the KB stage displacement or directly assigned the geometry from the nearest-neighbor reference configuration. Here we discuss the extrapolation and nearest-neighbor geometry calibration reduction routines as they are implemented in the *Data Analysis Workbench* (*DAWN*) software (Filik *et al.*, 2017 ▸). Our results demonstrate that uncertainties in the diffraction geometry make the extrapolation approach less accurate than the approximation introduced by the nearest-neighbor approach if a suitably dense grid of reference beam positions is calibrated. We finally present a systematic characterization of the diffraction resolution achieved by the DIAD instrument for different area-detector arrangements and grid densities of reference beam positions.

## 2. Methods

### 2.1. Diffraction-geometry calibration

At synchrotron sources, diffraction geometries are adjusted to work with complex instrument constraints leading to tailored configurations for individual experiments. In contrast to conventional laboratory diffractometers, the choice of a detector position, sample type and sample positioning is unique to each experiment. The most accurate assignment of the observed scattering momentum is then achieved via instrument calibration with a known standard sample performed at the start of every experiment. This problem led to the accurate investigation of different calibration strategies for a diffraction geometry using flat-panel detectors (Hart *et al.*, 2013 ▸). Ideal background models are now well established (Caglioti *et al.*, 1958 ▸; Cheary *et al.*, 2004 ▸). The calibration of a single scattering image can broadly be broken down into the following steps: point finding, outlier rejection, indexing and diffraction-geometry optimization. An exhaustive discussion of each of these steps is beyond our scope. Here we focus on the key areas required to understand the challenges introduced by the movement of the beam implemented at DIAD.

**Figure 2:**
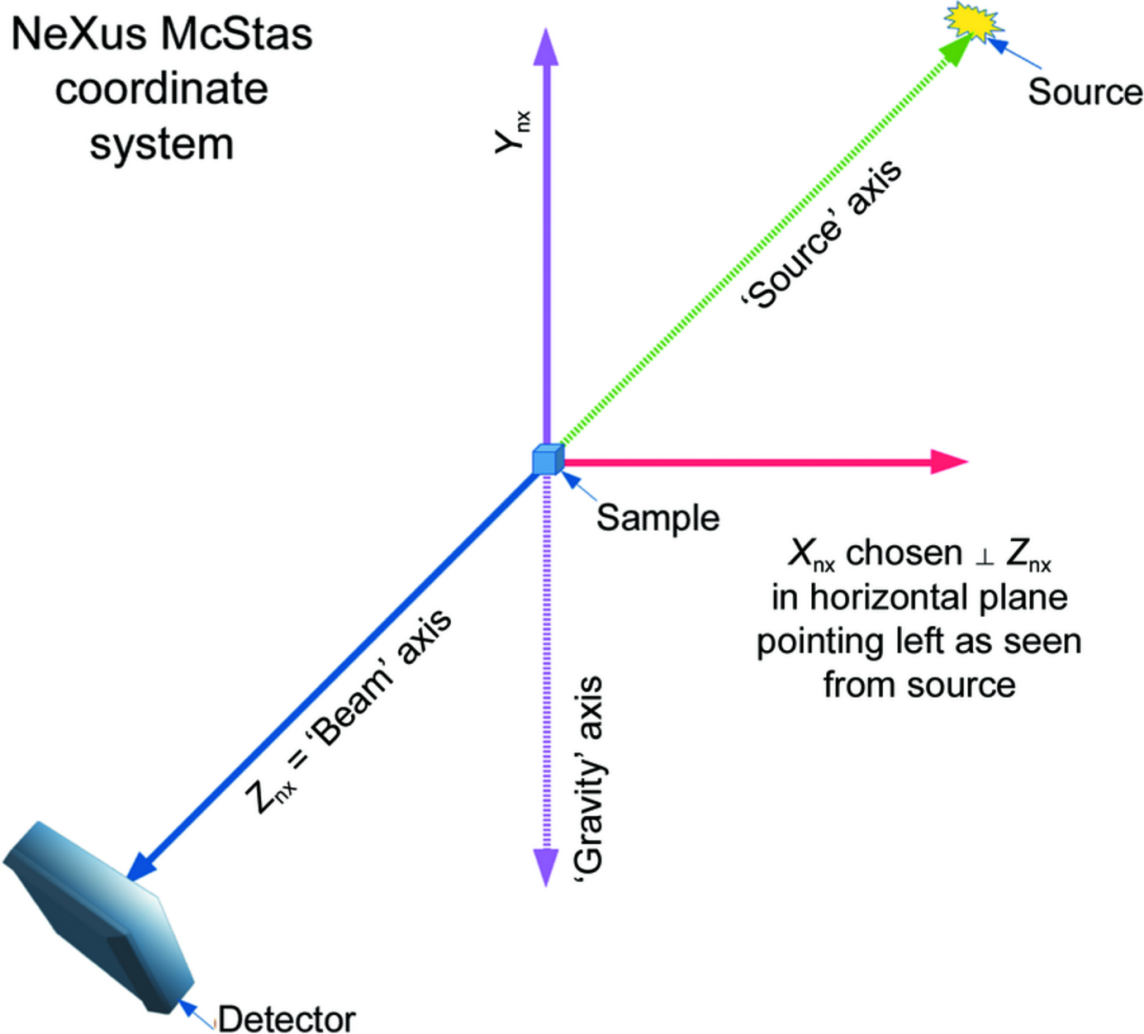
Coordinate reference system at the DIAD beamline. The *McStas* coordinate system convention is shown, with the *y* axis opposite the gravity direction and the *z* axis aligned with the imaging beam. The area detector is shown in the real-space configuration and the observed portion of the Ewald sphere is mapped in the reciprocal space. Taken from Bernstein *et al.* (2020 ▸), published under the terms of a Creative Commons Attribution (CC-BY) Licence.

In the DIAD beamline, we fine-tune harmonic rejection and the monochromator mirrors’ orientations (*i.e.* pitch and roll) to align the imaging and diffraction incident beams with the axes of rotation of the tomography stage, which is otherwise kept stable over time in position and direction thanks to a set of high-precision active step motors. The intersection of the imaging beam with the rotation axes then defines the origin of the beamline reference system as co-located with the sample position. Bragg’s law describes the kinematic diffraction, which relates the scattering angle 2θ with the structure symmetries of the periodic arrangement of atoms in a crystal. Constructive interference is observed for 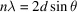 (Bragg & Bragg, 1913 ▸), where *d* is the interplanar distance, 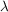 is the wavelength, *n* is the reflection order, and the scattering angle 2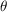 is defined as the angle between the incident beam and the scattered beam direction toward the detector. The sample is then the origin of the reference system for the definition of the diffraction geometry (He, 2009 ▸). Hereinafter we adopt the *McStas* standard to describe the reference system (Fig. 2 ▸), although alternative options are also commonly used (Bernstein *et al.*, 2020 ▸; Lefmann & Nielsen, 1999 ▸; Willendrup *et al.*, 2004 ▸, 2014 ▸; Willendrup & Lefmann, 2020 ▸, 2021 ▸). The *McStas* system describes the incident-beam vector as the cardinal direction 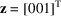 and assigns the 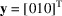 direction as opposite to gravity. The detector is then described by its normal direction 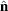, pointing away from the sample, and the vector 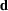 at which the detector plane intercepts the direct beam.

The moving-beam geometry of DIAD yields inevitable definition discrepancies. To ensure that both imaging and diffraction centers of scattering coincide at the sample position, the diffraction incident beam’s real direction has a shallow angle of ∼0.2° relative to the imaging incident beam, which is used to align the beamline optics and registers the beamline Cartesian reference system. The real-space detector-pixel position, 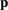, and scattering-momentum vector, 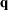, are related through the Ewald sphere construction (Ewald, 1921 ▸). In the Born kinematic approximation of elastic scattering,
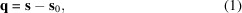
where 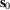 and 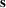 are the directions of the incident and scattered rays, respectively, and 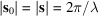. Hence, in the case where the detector normal unit vector, 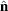, is not orthogonal to the propagation direction of the scattered beam, 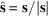,
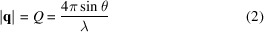
and
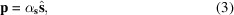
with
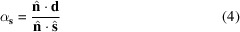
the distance from the scattering origin to the scattered ray’s intercept with the detector plane (Fig. 2 ▸).

During the point-finding step, *i.e.* identification and location of Bragg reflections, the area detector is mapped into the reciprocal space according to the observation of the Debye–Scherrer cones by the corresponding diffraction rings from a powder sample, or the reflection spots from a single-crystal sample. A NIST certified reference material is used to ensure the lattice parameters are known with high accuracy, with the assumption of a cubic structure.

A full calibration of the diffraction geometry requires the solution of seven degrees of freedom: the photon energy of the incident beam (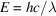), two coordinates of a point in the detector plane mapped into the reciprocal space, the sample-to-detector distance and three Euler angles that define the orientation of the detector plane. The models for calibration vary depending on sample type (*e.g.* single crystal versus powder), parameterization of the detector geometry [beam-center intercept versus point of normal incidence (Kieffer *et al.*, 2020 ▸)], and whether point- or ellipse-based refinement of the diffraction rings is used. Ellipse-based fitting routines require powder reference standards and full rings to be visible on the detector to limit the uncertainties of the ellipse parameters (Hart *et al.*, 2013 ▸). DIAD’s dual-technique design limits access to small-angle scattering and the azimuthal range to at most slightly less than 90°. The imaging apparatus, the scintillator housing and rail optics, and the collision protection systems [Fig. 1 ▸(*A*)] prevent the diffraction detector from being placed directly downstream of the beam path whilst capturing imaging data. This limits the azimuthal angular region available for calibration, as well as for measurement. Partial ring position refinement makes it difficult to decouple the detector distance from the energy and beam center, especially at large tilt angles. Hence, the photon energy of the instrument is measured independently of the final detector position.

### 2.2. Challenges from the moving-beam diffraction geometry

**Figure 3:**
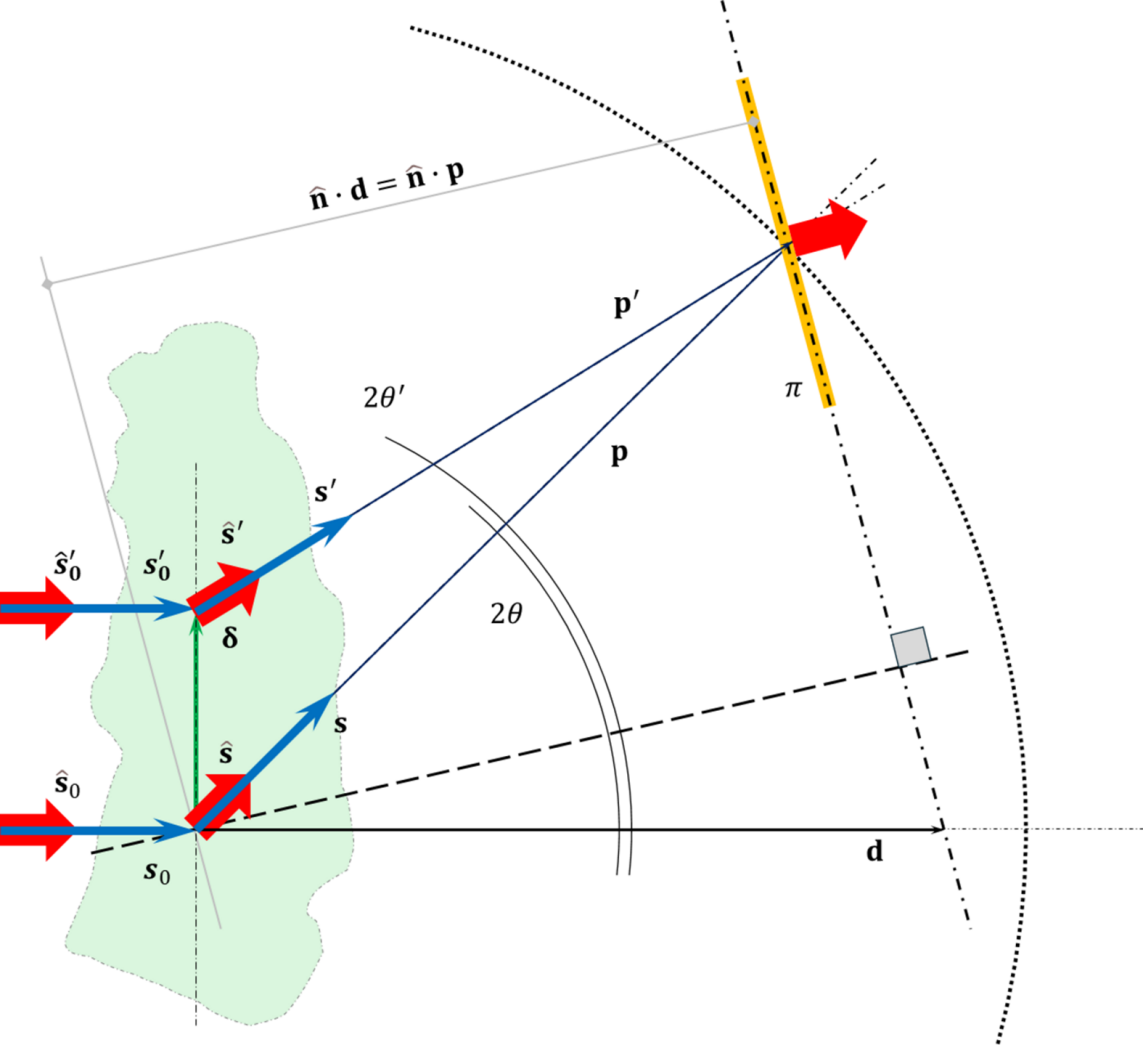
The moving-beam diffraction geometry. The beam (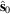) incident on the sample (green shadow) is shifted by **δ** to 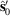 while the detector position and orientation is constant (yellow plane, 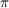). Although the detector distance **d** from the sample is not affected, the detector pixels will experience a relative change of their position from **p** to **p**′ because of the alteration of the diffraction geometry. For a given detector pixel, the scattered vector, 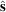, changes to 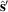, affecting the angle relative to the incident beam (*i.e.* 2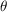 to 2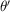). This causes a shift in the recorded *Q* parameter.

Changes in the beam position alter the origin of the diffraction-geometry reference system, affecting the scattering-momentum vector observed by any detector pixel (Fig. 3 ▸). The inaccurate calibration of the diffraction data alters the interpretation of direction-dependent measurements, such as strain and texture, and the interpretation of peak-broadening effects in the azimuthally integrated data due to the errors of the beam center. The movement of the diffraction beam invalidates the use of a single calibrated reciprocal-space mapping of the pixels.

A translation **δ** of the reference-system origin causes translation of the scattering vector 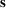 associated with a given calibrated detector-pixel position 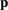. Hence, after translation, the detector-pixel position in the translated reference system will be
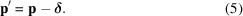
The new scattering vector,
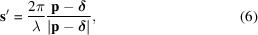
determines, then, the new scattering momentum 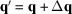, with the detector-pixel calibration error (see Appendix *B*[App appb] for the derivation)
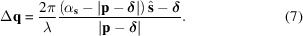
A robotic arm allows for adjusting DIAD’s detector configuration in space (Reinhard *et al.*, 2022 ▸). In particular, the detector’s outward normal 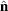 and the sample-to-detector distance *r* can be specified in a spherical reference system as
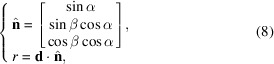
with 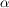 and 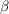 as the Cartesian angles within the *McStas* reference system.

**Figure 4:**
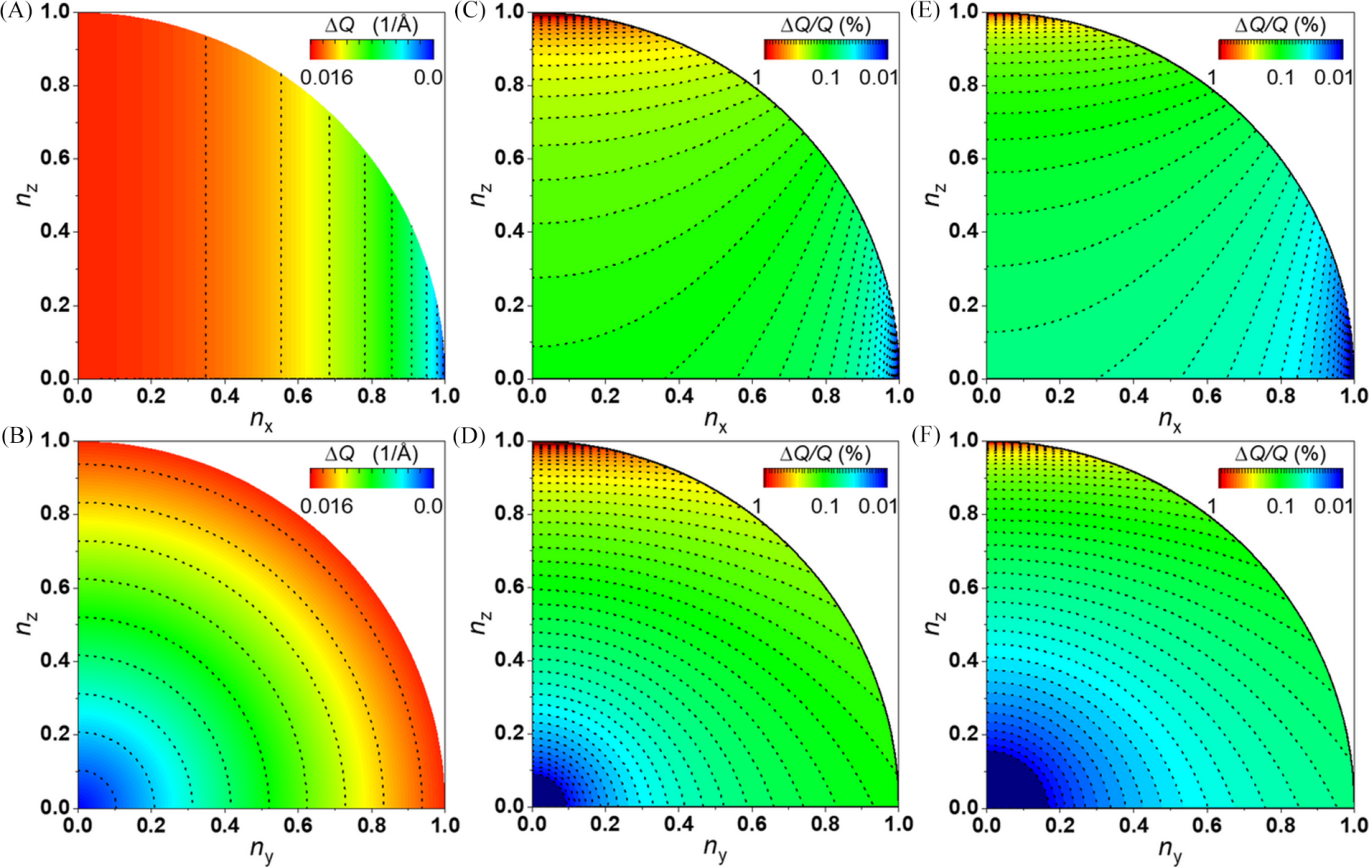
Effect of moving the diffraction beam on the scattering observation. Scattering-momentum error 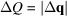 for a detector pixel on the first sphere octant arising from a 0.5 mm beam offset along *x*. The error is shown for the sample-to-detector distances of (*A*)–(*D*) 330 mm and (*E*), (*F*) 600 mm, assuming 20 keV radiation (0.619924 Å) and the *McStas* reference system (*i.e.* the incident radiation is parallel to *z*). (*C*)–(*F*) The relative error 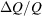 is independent of the radiation energy. The data in the upper and bottom rows differ only in the projection bases [(*A*), (*C*) and (*E*)] *n*_*x*_–*n*_*z*_ and [(*B*), (*D*) and (*F*)] *n*_*y*_–*n*_*z*_, respectively. The scattering momentum *Q* varies from 0 to ∼14 Å^−1^ with *n*_*z*_ from 1.0 to 0.0 (*i.e.Q* is independent of *n*_*x*_ and *n*_*y*_).

Fig. 4 ▸ compares the effect of a 0.5 mm diffraction scanning beam offset in the *x* direction (*i.e.* horizontal, normal to the beam), which is about half of the imaging FOV, for a point detector on the forward-scattering sphere octant with the sample-to-detector distances of 330 and 600 mm. The change of azimuthal angle causes a scattering-momentum error proportional to the component of the beam offset, **δ**, orthogonal to the pixel detector position, 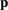, relative to the sample-to-detector distance, 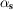. Although the error diverges in the small-angle region, in the wide-angle region away from the direct beam the relative scattering-momentum error is ∼0.1% (*i.e.*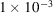) or ∼0.05% (*i.e.*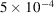) for the sample-to-detector distances of 330 and 600 mm, respectively.

Additionally, given that in the optimal configuration the pixel sensors in a flat-panel detector are arranged on a surface tangent to the sphere centered at the sample, the projection error introduced at each pixel strongly depends on the combined effect of the beam offset and each pixel’s location on the detector. This effect magnifies with the detector orientation such that none of the sensors are tangential to the sphere. It is, therefore, imperative to assign the correct detector orientation to avoid any diffraction-geometry aberration and measure accurately the magnitude and direction changes of the probe.

Compared with traditional strain gauges that can achieve a resolution of about 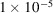, diffraction methods are expected to offer a strain resolution of one order of magnitude higher, *ca*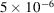, which is suitable for accessing information on the structure deformation. Hence, the beam offset causes an error significantly larger than the resolution required for mechanical strain analysis in materials. A new calibration protocol is required to recover the expected accuracy, making the moving-beam diffraction geometry distinct from established alternatives.

### 2.3. Calibration of the moving-beam diffraction geometry

The accuracy of any diffraction information computed from area-detector observations, *i.e.* structure and microstructure properties, relies on the ability to resolve the scattering angles and energy of the incident radiation with high reliability. Methods for high-resolution calibration of area detectors leverage multiple images or complex adjustable detector positioning to improve estimates of scattering-geometry parameters (Hart *et al.*, 2013 ▸; Horn *et al.*, 2019 ▸; Wright *et al.*, 2022 ▸). Here we use a grid of diffractograms to overcome the ambiguities produced by the rotation of the conic section about the beam axis and the full detector orientation for a single reference image. First, we calibrate a single image to be used as the seed for further automatic calibrations. The data collected for a grid of diffraction-beam offsets with known rigid translations of the focusing are then independently calibrated. The real-space configuration of the detector can be reconstructed on the basis of the changes observed in the detector origin estimated from the changes in pixel position of the diffraction rings. The effect of the offset of the incident beam can then be determined by simple extrapolation.

In contrast to stationary experiments, the correct seed detector configuration is key to the accuracy and confidence of the extrapolation procedure. The extrapolation process assumes that the beam orientation of the incident beam is independent of the beam position. At DIAD the rigid translation of the focusing KB mirror with two high-precision step motors realizes the offset of the diffraction probe. These motions are built orthogonal to the beamline general design *z* axes, which are generally not aligned with the diffraction or with the imaging beam. This causes the moving-beam operation to work with unavoidable unknown geometric alterations of the incident beam in addition to the idealized pure translation. Because of these constraints and the overall approximations involved with the extrapolation process, our default approach is to rely only on the directly calibrated grid of datasets.

Nearest-neighbor routines assign to each collected dataset the calibrated instrument geometry associated with the KB position in the setup grid that is closest to the KB position used during the experiment. We assume then that the corrected calibration of the beam movement remains valid regardless of the sample, as long as there is no change of diffraction detector and beamline optics configuration (*i.e.* orientations and positions). Moreover, centering of the sample on the tomography-stage rotation axis ensures that sample movements away from the calibrated position can be reduced to less than the resolution of the imaging branch, 1.6 µm, *i.e.* three times the imaging pixel size of 0.54 µm.

### 2.4. Application of the moving-beam geometry in experiments

In contrast to traditional Bragg–Brentano and Debye–Scherrer geometries, which are optimized for flat-plate and capillary phase-homogeneous samples, respectively, the moving-beam geometry is optimized for the study of samples that are spatially phase and morphology inhomogeneous. The data collection in transmission mode enables the use of non-standard sample geometries, which is essential for the core purpose of the moving beam: to spatially resolve structural variations across macroscopically inhomogeneous systems. The moving-beam geometry requires samples to have a microstructure such that the crystal size yields a powder-like scattering from a probe volume of a few micrometres. Hence, at DIAD we use a KB mirror system to dynamically adapt the beam focus according to the crystal size and the required spatial resolution of different samples. DIAD offers three standard setups with a beam focused to areas of 5 by 15 µm, 25 by 25 µm or 50 by 50 µm, which are suitable for a wide range of fields of study, including mechanical engineering, geo­sciences, energy materials, and medical and biological systems. The trade-off for this limitation is the key advantage of the moving-beam geometry compared with more traditional geometries: the opportunity to probe different well defined regions of the samples without their perturbation by sample movement. This opens access, particularly, to the study of processes affected by time-dependent alterations of the chemical components’ concentration in a liquid environment, where sample motion would cause re-mixing and so alter the time-evolution process. Examples are the study of crystallization phenomena from liquid or melted liquid baths, metal oxidation, degradation of ceramic and biological materials exposed to aggressive liquid environments (*e.g.* salty water and body-fluid simulants), and chemical reactors.

With the region to be probed not being stable, the geometry calibration with a standard capillary geometry becomes impractical, and a flat-plate sample in transmission mode aligned orthogonally to the imaging beam path is left as the best average approximation of a generic-shape macroscopic sample. The calibration on a plane orthogonal to the imaging beam path provides the best balance between induced aberrations (*e.g.* sample shift, absorption, sample geometry *etc*.) and access to local information. In particular, the effective center of scattering is shifted compared with the calibrated position, either because of the sample shape or because of absorption inhomogeneity. As the actual center of scattering can change across the sample itself or sample by sample, the calibration for the 2D plane standard provides a reference point for sample-dependent corrections to be applied at the stage of line-profile analysis. Our typical calibration standards, with nominal thicknesses of 0.5 mm, offer reasonably accurate flatness despite challenges in powder packing. While static samples inherently suffer from reduced statistical averaging and increased preferred orientation effects compared with spinning geometries, these limitations are well characterized and do not significantly impact geometry calibration. However, they do pose challenges for quantitative diffraction analysis, particularly in texture-sensitive applications. Unlike for traditional diffractometer instruments, the typical sample thickness is limited to less than 1 mm to fit in the imaging FOV, and most of the sample geometry and composition aberrations can be corrected on the basis of imaging radiography or tomography information. Indeed, imaging provides not only the information for a possible full absorption correction but also a measure of the sample shift from the calibrated center of scattering with a resolution of 0.5 µm.

### 2.5. Diffraction calibration configurations

Experimental constraints dictate the choice of detector position, standard reference material and beam energy. Here we characterize the calibration accuracy and precision of the most common combinations to assess the reliability of the moving-beam diffraction geometry of normal experiment visit operation.

#### 2.5.1. Detector positions

**Figure 5:**
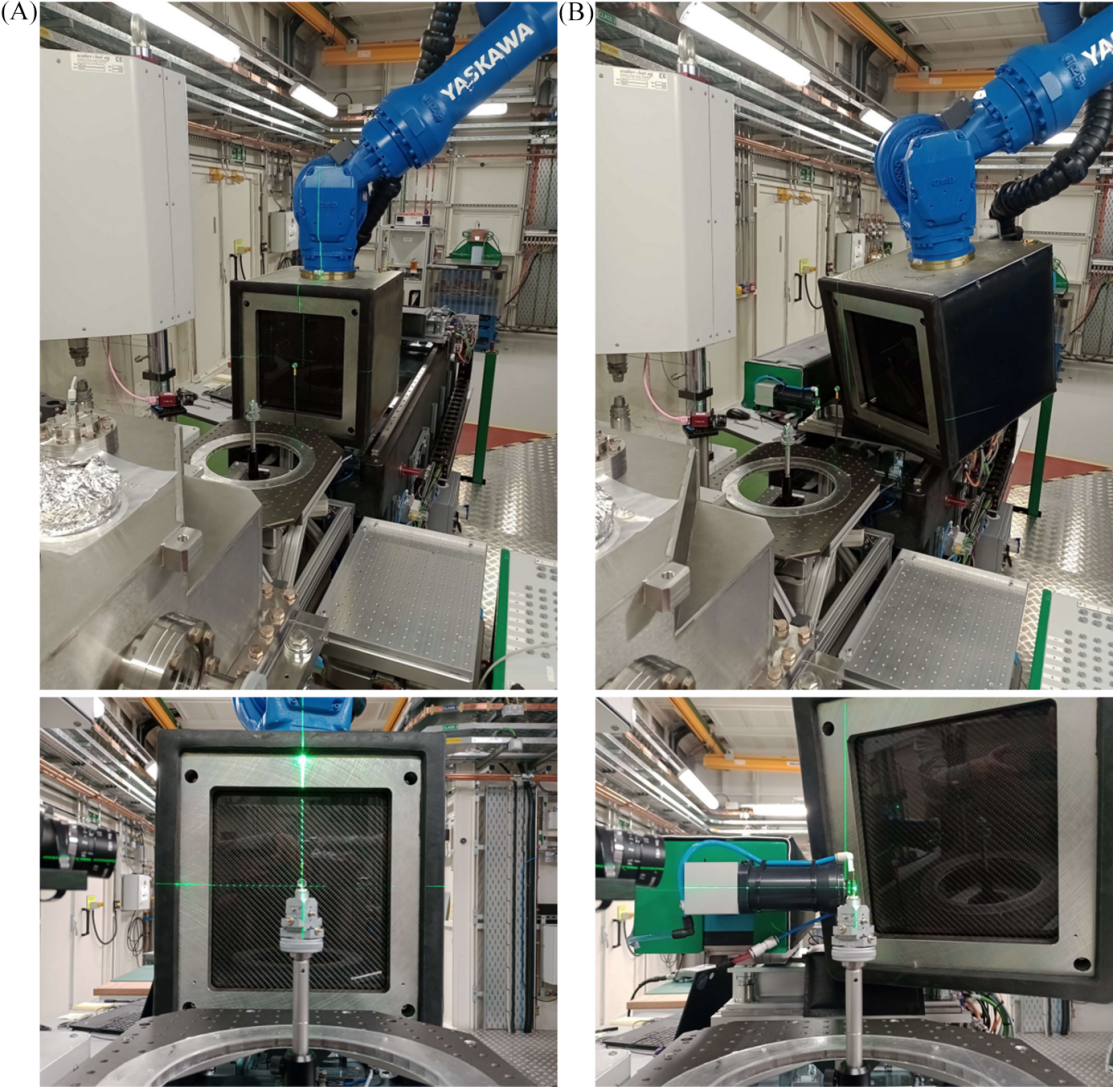
Photographs from the DIAD experimental hatch showing the detector positions (*A*) with the conventional transmission detector geometry and (*B*) tilted relative to the incoming beam.

The spherical system previously presented by Reinhard *et al.* (2022 ▸) specifies the position and orientation of the detector. In this system, the center of the detector plane is imposed to be orthogonal to the direction of scattering, making the diffraction-geometry calibration simpler. We assess the quality of the calibration for two characteristic detector positions: (i) the conventional transmission detector geometry and (ii) tilted relative to the incoming beam (Fig. 5 ▸). Whereas, with the conventional transmission geometry, the incoming beam is orthogonal to the detector plane and incident at the central pixel of the panel, with the detector tilted relative to the incoming beam, the detector panel is also shifted inboard.

With the conventional transmission detector geometry, the sample-to-detector distance is *ca* 330 mm and the pixel at the center of the 2D sensor panel is orthogonal to the scattering direction. Otherwise, with the detector tilted relative to the incoming beam, a sample-to-detector distance of *ca* 300 mm and a central detector pixel at α = 27°, β = 15° and γ = 0° are chosen. This position maximizes the ring section covered while still permitting imaging data to be acquired, representing realistic experimental conditions.

#### 2.5.2. Standard samples

The standard reference materials 660b LaB_6_ and 674b CeO_2_ from NIST are chosen to ensure a sufficient number of rings to cover a large proportion of the detector plane and provide a suitable number of crystals to form complete powder rings without introducing excessive peak broadening. The cryo-cooled Si(111) double-crystal monochromator of the DIAD diffraction branch (DCM-1) operates in the energy range from 7 to 38 keV. At 25 keV and in the conventional transmission detector geometry, the first six and five innermost reflections of SRM 660b LaB_6_ and SRM 674b CeO_2_, respectively, are fully accessible. At energies higher than 25 keV, the rings of SRM 660b LaB_6_ come too close to ensure easy imaging processing, so SRM 674b CeO_2_ is preferable.

**Figure 6:**
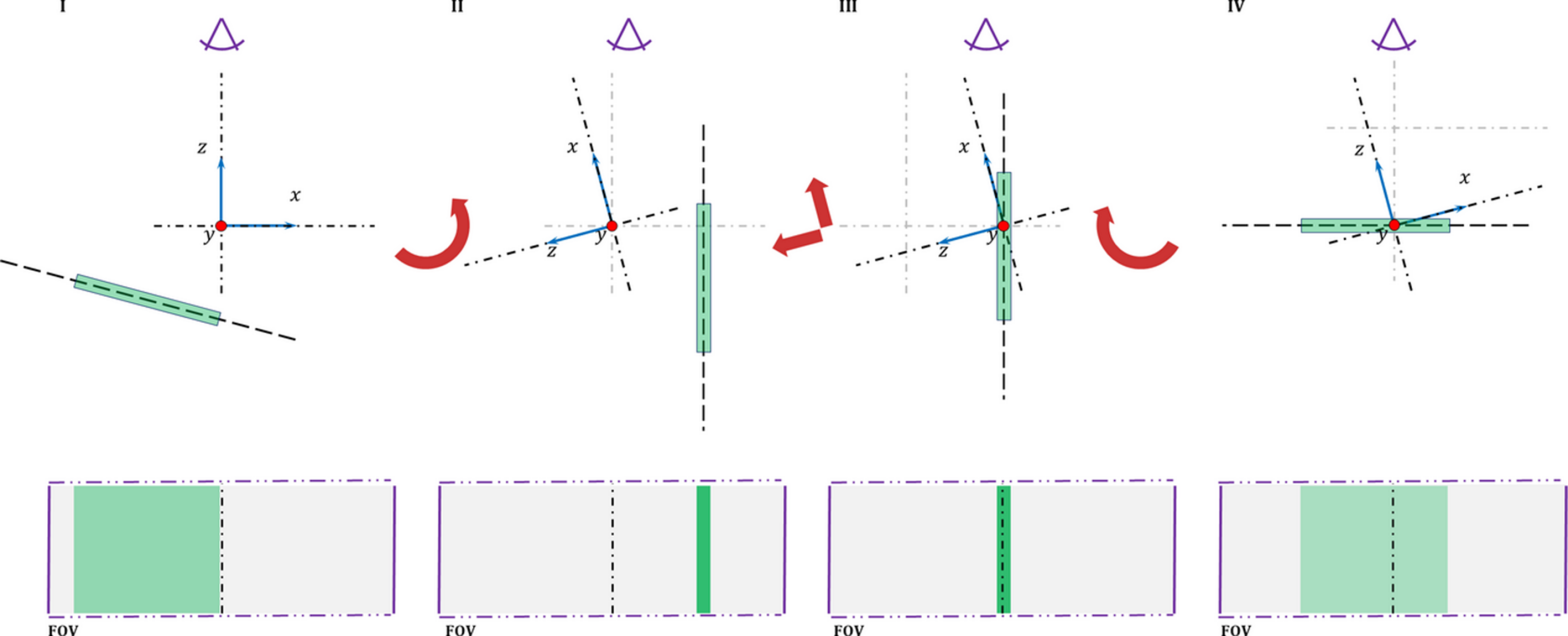
Sample alignment with the tomography axes of rotation. Samples are aligned with the GTS axes of rotation, exploiting the fact that the *x*–*z* motor is mounted above the rotation motor. After the flat-plate sample is oriented orthogonal to the FOV plane (I 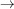 II), it is shifted to the center of the FOV (II 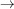 III), which is aligned with the axes of rotation at the start of the beamline setup. Finally, a 90° rotation is applied to bring the plane of the sample orthogonal to the beam stream (III 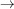 IV).

Calibration samples are prepared in a 0.3 mm-diameter borosilicate capillary and on a 0.5 mm-thick ∼2 cm-wide polyimide flat plate. Although the capillary form allows spinning to improve sampling statistics, the tubular shape limits the beam offset to a single scanning direction. On the other hand, the flat-plate shape enables the beam to offset along two independent scanning directions; but without the sample spinning the number of crystals contributing to the scattering signal is limited to those lying within the beam footprint through the sample. Samples are aligned with the axes of rotation using the imaging camera and the tomography stage. Although the samples are manually aligned with the gravity axes, the plane of the flat-plate holder is registered orthogonal to the imaging beam, exploiting the fact that, for the general tomography stage (GTS), the orthogonal *x*–*z* displacement motor is mounted on top of the rotation motor. Flat-plate samples are tilted until they are parallel with the imaging beam and then turned by 90° to be about orthogonal to the incident beams (Fig. 6 ▸).

#### 2.5.3. X-ray energy

The beam energy is tailored to the experiment requirement on the basis of pre-recorded Bragg angle and crystal-to-crystal distance tables. Despite this, the beam energy is estimated at the start of each new experiment session because it plays a crucial role in the diffraction-geometry calibration as well as in the analysis of any diffraction data

Given that at the start of the beamline setup the beam position at the sample is usually not yet synchronized with the KB motion, we use the diffraction signal from a standard flat-plate sample and the conventional transmission detector geometry to measure the energy of the incident beam. This position provides full ring coverage on the detector, and the direct beam lies close to the center of the detector. Several full reflections can be detected with cone half opening angles of 2θ ≤ 19.5°. The downstream configuration also minimizes the errors caused by uncertainty of the beam position and the ambiguity between energy and sample-to-detector distance, both affecting the observed radius of the diffraction rings by the same factors. Samples are mounted and aligned with the rotation axis of the tomography stage before positioning the diffraction detector to exploit the imaging beam and detector camera. The beam-energy value is optimized to match the pattern of the full diffraction rings on the basis of the known spacing dictated by the standard material and an empirically optimized seed for the diffraction-geometry and beam-energy parameters. It is well known that the results of automatic optimization routines are affected by the seed guess (*i.e.* the closer the seed is to the true solution, the more likely the optimization routine will converge to the latter).

Although this process allows for easy and time-efficient calibration of any energy ahead of normal operation, high-accuracy beam energy is otherwise achieved by detecting the absorption edge of a standard foil material. The Bragg angles of the DCM crystals are scanned and the integrated fluorescence intensity from the foil is measured across the imaging camera. The DCM is then set to the angle corresponding to the absorption edge of the foil (angle of maximum fluorescence). At DIAD, we use Zn, Mo and Sn foils to independently identify the DCM angles for 9.66, 20.00 and 29.20 keV energies, respectively.

## 3. Results and discussion

### 3.1. Single-image calibration accuracy

The accuracy of the moving-beam geometry is particularly sensitive to the accuracy of the reference calibration. Hence, uncertainties arising from the identified ring locations, limited ring coverage and large diffraction spots can all degrade the quality of the correction.

**Figure 7:**
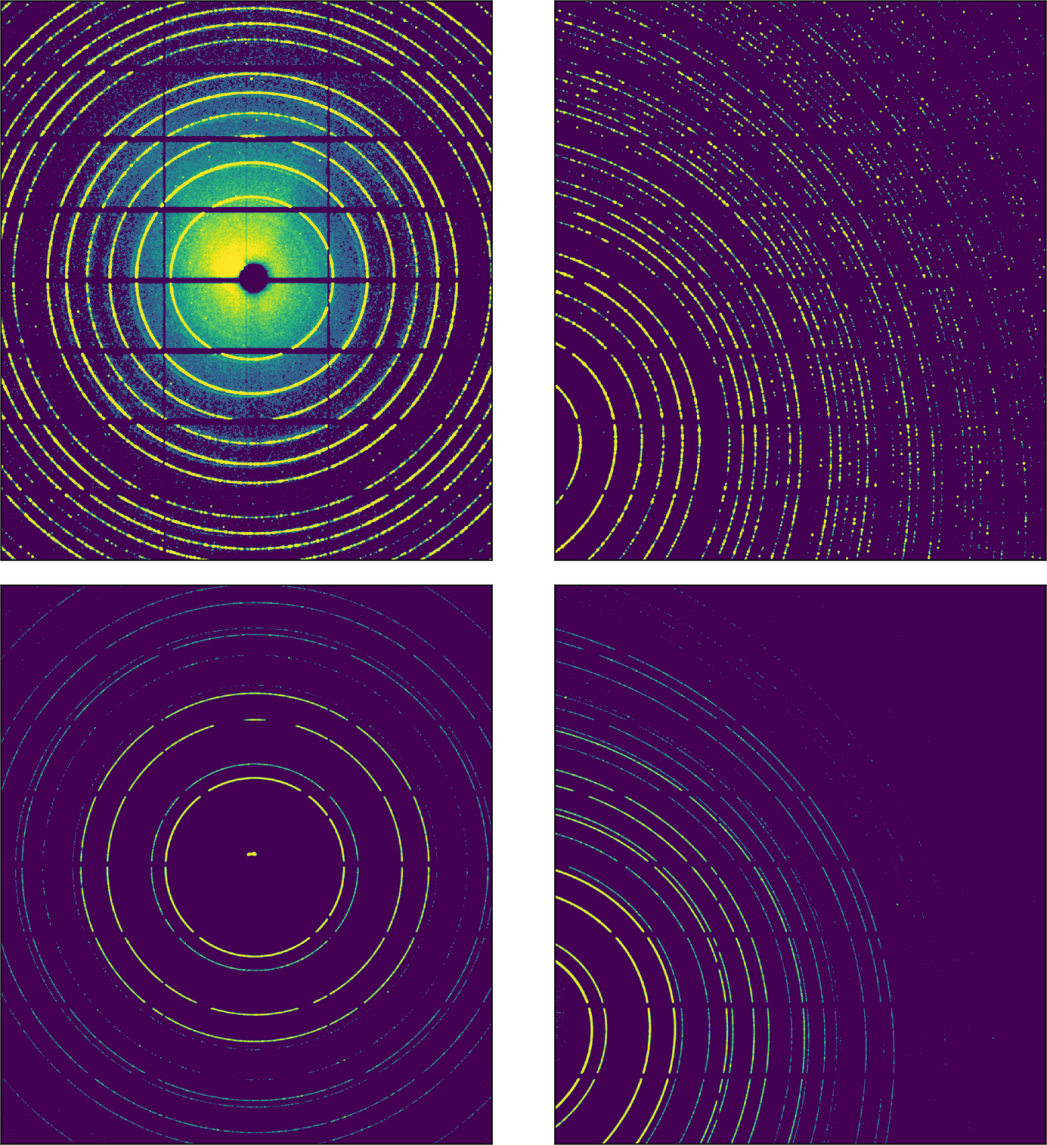
Diffractograms from SRM 660b LaB_6_ (upper row) and SRM 676b CeO_2_ (bottom row), for the conventional transmission detector geometry (left column) and the detector tilted relative to the incoming beam (right).

The diffraction-beam energy is calibrated at 25 and 30 keV with SRM 660b LaB_6_ and SRM 674b CeO_2_ samples, respectively. Between detector-position movements the diffraction branch optics are not altered to ensure that the energy measurement made with the conventional transmission detector geometry remains valid for the tilted relative to the incoming beam detector position. Data are collected from both sample types using both detector positions (Fig. 7 ▸). Diffraction data are collected using the Pilatus2M CdTe detector with a pixel size of 0.172 mm. A vertical scan of 120 points is taken on both samples using the KB *y* movement only to ensure consistency between sample alignment, *i.e.* without sample movement. Acquisitions with the capillary samples are conducted with the stage rotating to maximize data quality, *i.e.* powder statistics. On flat-plate samples, an additional map of 121 grid points is measured per detector position using the KB movement orthogonal to the beam path. This consists of a 121 × 120 point grid centered with the FOV of the imaging branch and covering a range of 1.2 × 1.2 mm with step sizes of 120 µm per KB motion.

A single-crystal dataset is also studied to improve the accuracy of the detector roll angle, which plays a significant role in the correction procedure. The GTS rotation axis that holds the samples is specified as 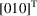, assigning the tomography-stage rotation axis anti-parallel to gravity consistent with the *McStas* coordinate system. A sphere of SRM 1990 Al_2_O_3_ is mounted on a 200 µm MiTeGen loop and aligned to the rotation axis of a 1005 Huber goniometer. During the acquisition, the crystal is rotated about the reference axis at an angular rate of 0.1° s^−1^ with a detector exposure time of 1 s. This ensures that individual Bragg reflections appear for multiple rotation steps, improving the reflection centroid refinement. In total, 16 datasets are investigated with the crystal orientated at multiple poses by manually rotating the sphere about the goniometer’s axis. Assessment of the rotation center in the tomographic reconstruction confirms that the rotation axis of the GTS is vertical in the imaging space and can therefore be used as a reliable reference for processing the single-crystal datasets over the 16 poses.

#### 3.1.1. Data analysis: calibration, reduction and fitting

Individual-frame calibration is performed using 500 detector-pixel points per diffraction ring. The radial region window to identify these points is computed on the basis of the list of reflections in Table 1 ▸ and a rough seed manual calibration. The reference orientation and detector geometry are manually adjusted to provide a visual agreement between estimated and observed ring projections in the 2D detector image. Although the seed provides only a first-approximation qualitative match, the calibration parameters are expected to resemble, unless there is a change of reference-system convention, the nominal configuration reported by the robotic arm that holds the Pilatus detector in position (Reinhard *et al.*, 2022 ▸).

The detector orientation of the initial seed is converted to the equivalent 0°-roll configuration to obtain consistent calibration estimates of the diffraction-beam offset frames. The scattering from a randomly orientated powder in a two-dimensional flat-panel X-ray detector results in the same Debye–Scherrer cones for different equivalent detector orientations. All these equivalent orientations differ by a simple change of the detector roll angle around the beam vector with the rotation centered at the beam center on the detector plane. Indeed, given the scattering cones share the same rotation symmetry axes, only two degrees of freedom among yaw (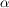), pitch (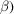 and roll (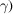 are independent. Any detector orientation is then fully described by the two angles 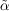 and 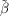. During individual-frame calibration, the beam energy is fixed at the value determined from the calibration with the conventional transmission detector geometry, ensuring that the observation accurately reflects the uncertainties in the detector configuration only (see Section 2.5.3 for more details).

The diffraction geometry is calibrated for each diffraction dataset. Traditional Bragg intensity profiles are then computed via azimuthal integration using the *DAWN* software (Filik *et al.*, 2017 ▸). At Diamond Light Source, the *DAWN* software offers the ability to perform on-the-fly data reduction, leveraging parallelization using a high-performance computing cluster. With *DAWN*, diffractograms are reduced to line plots using pixel-splitting integration. Other integration settings are adapted to the detector position and sample material as reported in Table 2 ▸.

Peak fitting is applied to the data using a compound model of a Voigt profile in combination with a flat background. Individual peaks are isolated according to the expected peak positions and a suitable search range of 7σ (*i.e.* ±3.5 FWHM) to ensure that additional correlations between the peak-center uncertainty and window size are negligible (Withers *et al.*, 2001 ▸). Peak fits are performed on several reflections with high multiplicity taken across various positions on the detector face. Peak-fit reflections for the two materials are provided in Table 3 ▸.

Single-crystal geometry calibration is performed using the *Diffraction Integration for Advanced Light Sources* (*DIALS*) software (Waterman *et al.*, 2016 ▸). In the refinement, we use the certified ruby-sphere crystal structure along with an initial estimate of the detector configuration. Prior to analysis, the DIAD standard NeXus file structure is reconfigured to conform to the NXmx standard format. The tilts of the two additional goniometer axes are determined from the analysis of the imaging datasets.

**Figure 8:**
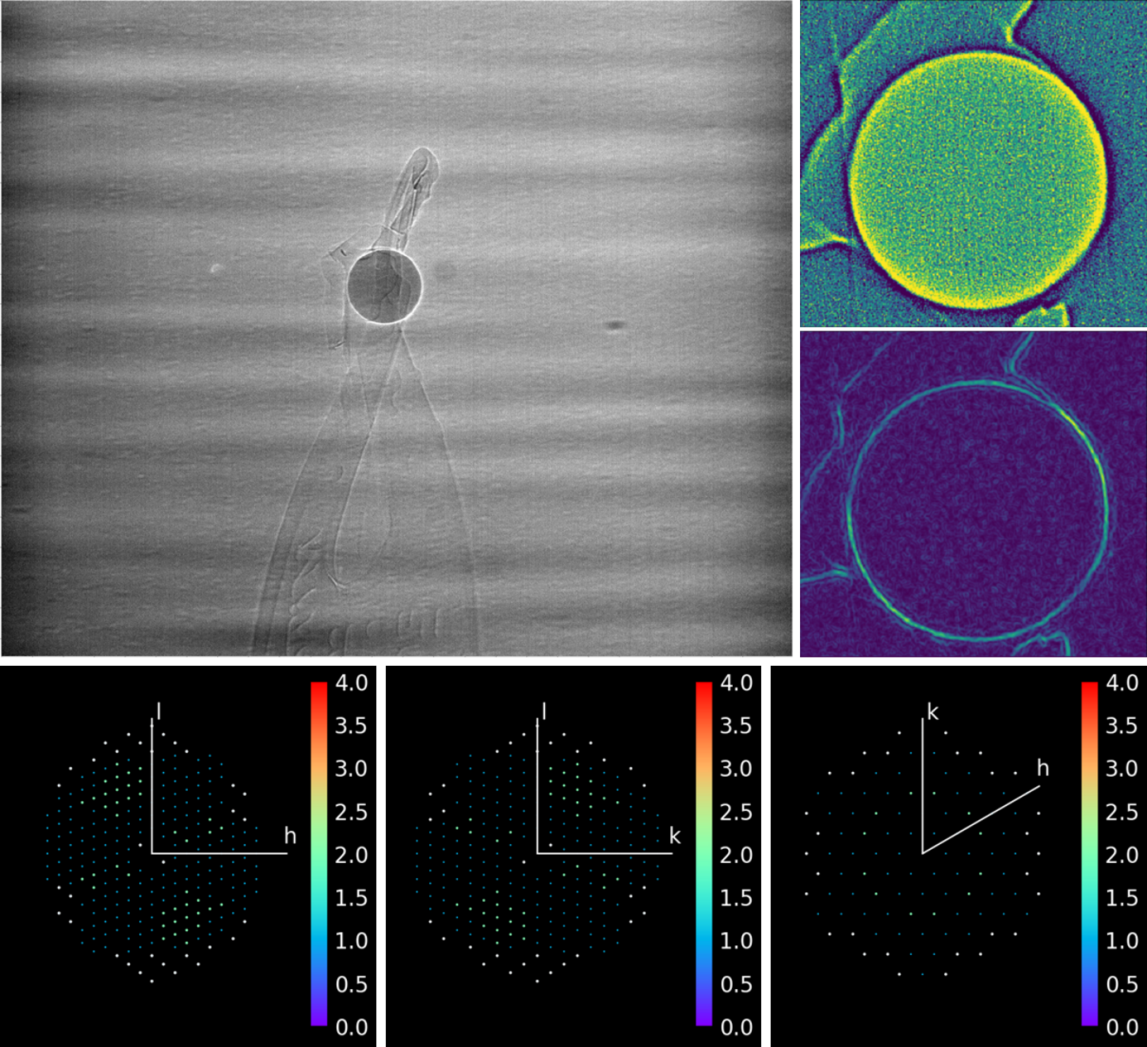
Single-crystal observation. Radiography snapshot at 0° (upper-left corner), and tomography reconstruction slice (top) and the same filtered (bottom) to measure the sphere centroid (upper right). Reciprocal-space reconstruction with *DIALS* along three different zone axes (bottom row).

A ruby sphere is aligned with the axes of rotation of the tomography stage and single-crystal diffractograms are recorded at constant step tilt rotations (Fig. 8 ▸). The small dimension of the crystal, below 0.2 mm in diameter, and the spherical geometry ensure that minimum aberrations affect the diffraction data. The constant beam cross section at different tilt angles removes the need for absorption correction. Results from *DIALS* analysis are in good agreement with expected values (see Table 4 ▸), such as the sample-to-detector distance of 365.447 mm and the *DIALS*-refined origin of the reciprocal space after the due conversions in space reference systems between *DAWN* and *DIALS*. The detector roll, pitch and yaw angles, 24.87°, −20.46° and −0.80°, respectively, are in good agreement with the corresponding values of 24.88°, −20.43° and −0.86° estimated by *DAWN* from analysis of powder diffraction data. However, the associated standard deviations (s.d.s) of 0.015°, 0.034° and 0.044° are significantly larger than the misorientation s.d. measured via *DAWN* refinements with known incident radiation energy, *i.e.* ∼0.007° [see Section 3.1.2 and Fig. 10(*C*) for more details].

Hence the single-crystal diffraction is less reliable than the powder diffraction process to measure the accurate detector-space configuration relative to the incident beam. This is reasonable as the single-crystal diffraction method is based on a much smaller number of reflection data points. Moreover, single-crystal diffraction is known to be affected by uncertainty over detector position, centroids of the single-crystal reflections, sample centering and consequent precession effects, and stability of the beam position. Conversely, the use of a large flat-area powder sample and the recognition of the diffraction rings from a 2D area detector provide a significant increase in statistics and accuracy of observations.

#### 3.1.2. Moving-beam calibration accuracy

**Figure 9:**
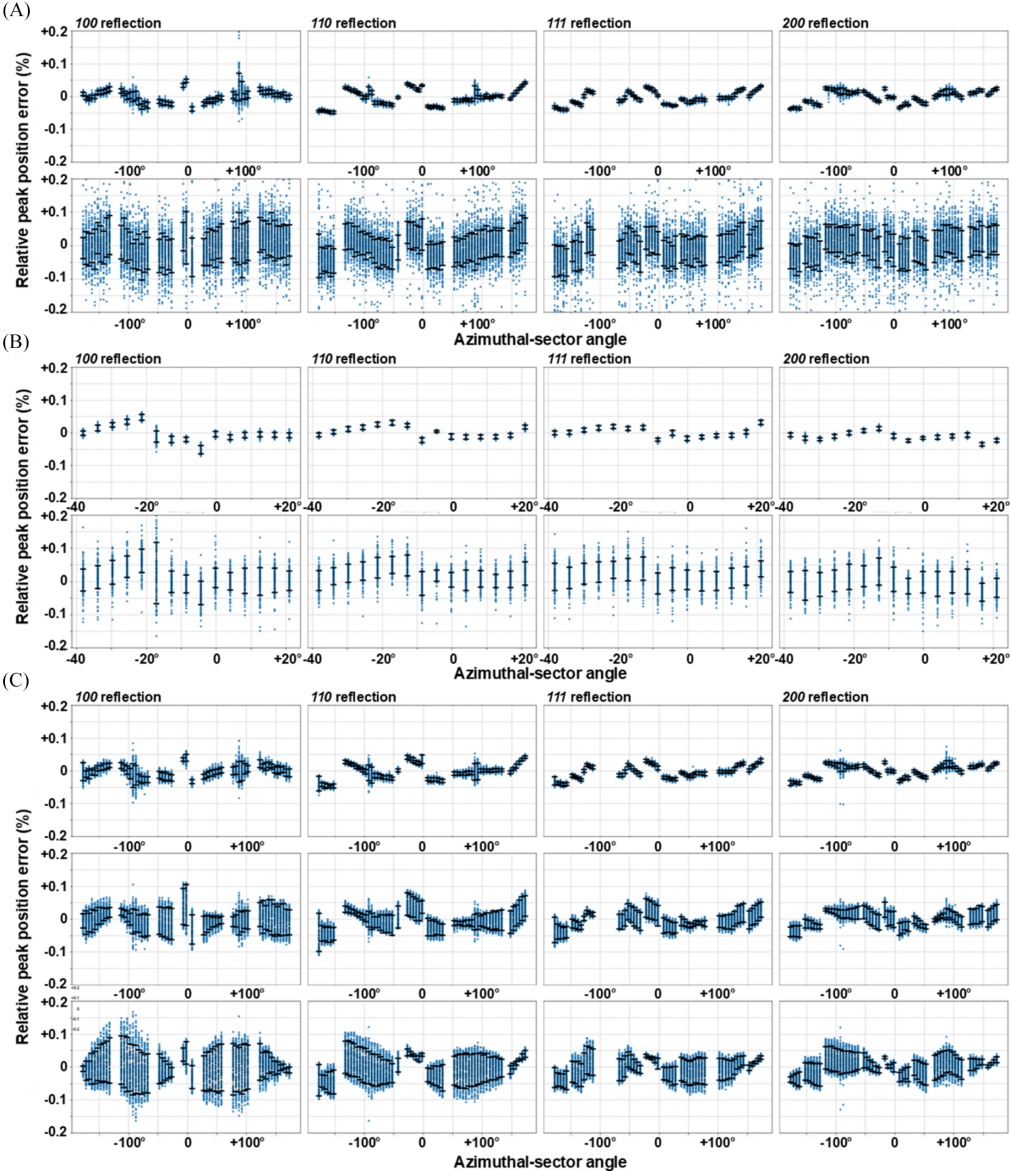
Calibration accuracy. Relative peak-position error for the 100, 110, 111 and 200 reflections with (*A*) the conventional transmission detector geometry and (*B*), (*C*) the detector tilted relative to the incoming beam. Diffractograms were integrated over a 5° sector of the azimuthal angle to assess precision and accuracy as a function of the projection angle across the detector panel. Diffraction measurements are repeated by applying a displacement in the gravity direction, *i.e.* tomography-stage *y* axes, to the capillary sample [first rows of (*A*) and (*B*)] and to a flat-plate sample in the normal-to-the-beam direction [second rows of (*A*) and (*B*), and all of (*C*)], *i.e.* tomography-stage combined *x* and *z* axes. In (*C*) the measurements are repeated for the same relative displacements of the center of scattering by moving the diffraction beam while the sample stays stationary. For this, the relative error is computed either using self-calibrated geometry [first row of (*C*)] or by extrapolating the geometry correction for the beam shift alone [second row of (*C*)] or the combined beam shift and relative detector tilt [third row of (*C*)]. A relative peak-position error of 0.05% corresponds to a relative interplanar-distance error of ~0.0065% for a 1.8953 Å *d*_0_ observed with a 25 keV X-ray beam.

From performing independent geometry calibrations for a two-dimensional grid of diffraction centers across the imaging FOV it is possible to investigate the assumption that the vector of the incident beam is stable across the full measurement area (*i.e.*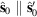). Fig. 9 ▸ shows the relative peak-position error estimated for the first four LaB_6_ reflections as a function of the sample [Figs. 9 ▸(*A*) and 9 ▸(*B*)] and diffraction beam [Fig. 9 ▸(*C*)] displacements. The plots reveal a recurring correlation pattern between the observed peak positions and the azimuthal-sector angle of observation. This pattern is probably due to alignment errors in the detector modules within the Pilatus2M panel. However, these errors fall well within the precision uncertainty associated with peak positions for each azimuthal-sector angle. The deviation remains within a 0.2% margin, regardless of the motion considered or any theoretical corrections applied to the diffraction geometry for the moving beam. Notably, the same increase of error is observed by moving either the diffraction beam or the flat-plate standard sample in the *x* direction, *i.e.* horizontal, normal to the beam. This demonstrates that the error is mostly caused by imperfections of the sample shape, *i.e.* nonconstant thickness, and possible tilt or bending of the flat-plate support, especially in the gravity *y* direction. Another significant source for the observed error is the tilt between the incident diffraction and imaging beams, resulting in a slight shift of the center of scattering along the beam path while scanning the flat-plate sample in the horizontal direction. Negligible improvement of results is achieved by compensation of the change of diffraction geometry, accounting for the extrapolation of the beam shift and relative detector tilt as a function of the displacement of the center of scattering [Fig. 9 ▸(*C*), second versus third row]. Indeed, the error more than doubles compared with self-calibrated datasets [Fig. 9 ▸(*C*), first row]. This demonstrates that the uncertainty of change of the diffraction geometry caused by the moving beam exceeds the accuracy of a single KB position calibration.

**Figure 10:**
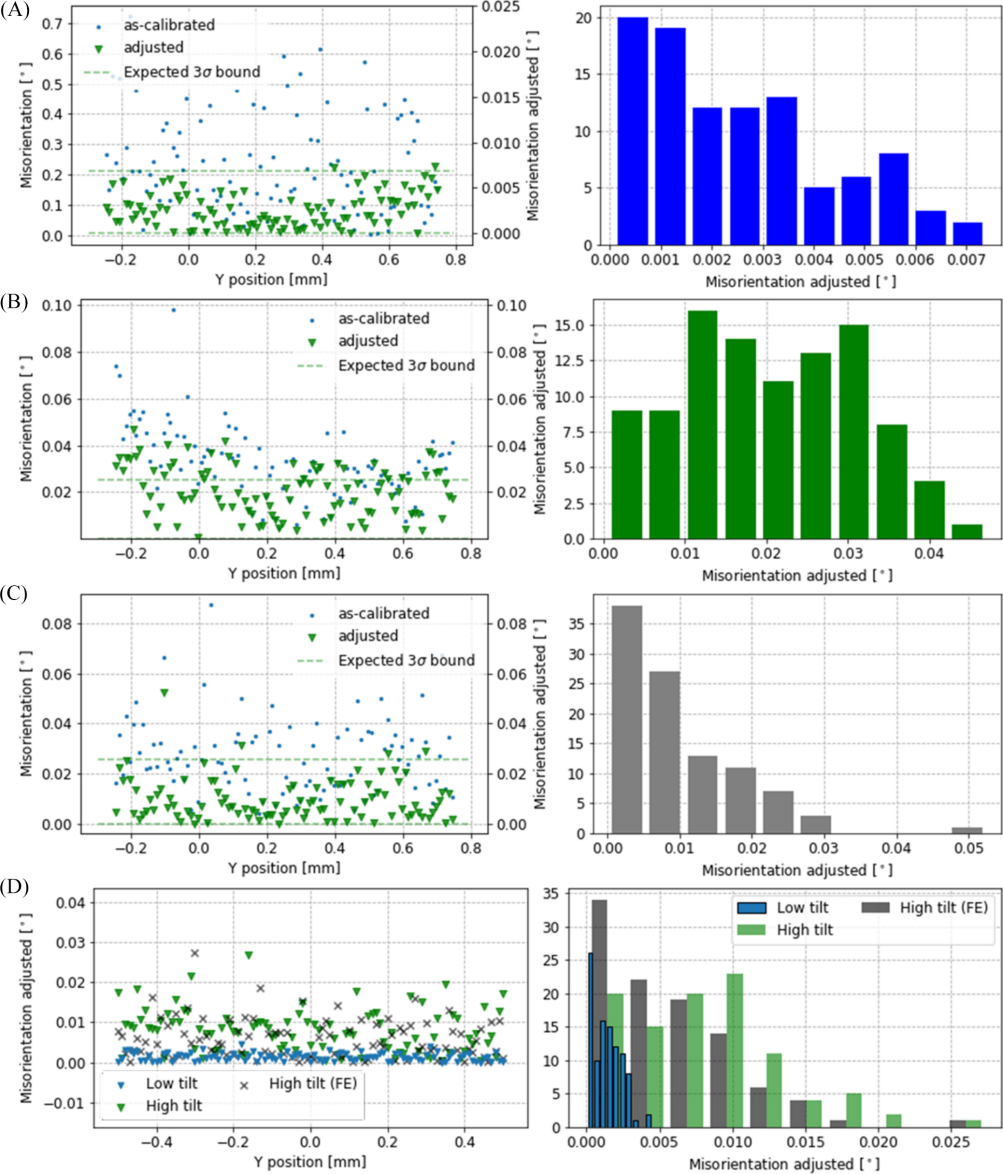
Detector misorientation. Individual diffraction calibration misorientation (left column) and corresponding frequency distributions (right column). The measurements from a LaB_6_ powder sample are repeated for different displacements of a capillary sample in the gravity direction while keeping the beam at constant KB position and with (*A*) the conventional transmission detector geometry and (*B*), (*C*) the detector tilted relative to the incoming beam. The calibrations were repeated including the energy (*A*), (*B*) as a free degree of freedom or (*C*), (*D*) fixed. (*D*) The misorientations measured with the same detector and sample configurations as (*A*)–(*C*) but also scanning the *y* axes with the KB position motion while keeping the sample steady. FE: front end.

The uncertainty of the detector orientation from a single diffractogram can be determined by taking the geodesic distance in 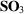 (Haslwanter, 1995 ▸; Novelia & O’Reilly, 2015 ▸), *i.e.* the group of all possible rotations about the origin of the three-dimensional Euclidean space, between the individual detector-orientation calibration and the corresponding orientation obtained from extrapolation through the orientation-matrix construction. In *DAWN* two angles are used to define the orientation of the detector plane. The uncertainty in the final orientation is then determined, representing the rotation matrices in the angle–axis formulation. The misorientation, ς, between two orientation matrices, 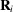 and 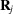, is computed directly from knowledge of the two orientations. However, because of the scattering cone symmetry, it is possible that two orientations display large differences while having equivalent diffraction signal. Hence, adjustment of one of the rotation matrices to account for this ambiguity is required. This is done by numerically solving
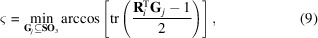
where 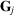 is the subset of the group of rotation matrices 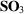 that produces an orientation of 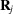 with equivalent diffraction signal. Given that the principal value range of 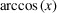 is confined between 0 and π, the misorientation between two diffractograms of a group is expected to follow a folded-normal distribution (Fig. 10 ▸, right column). The misorientation distribution between recalibrated diffractograms is shown in Fig. 10 ▸. The s.d.s derived from the folded-normal distributions agree with the uncertainties derived from the single-image orientation uncertainty [Fig. 10 ▸(*D*)]. This indicates that the diffraction incident-beam direction has no significant orientation deviation over the imaging FOV. Notably, no significant improvement is observed when constraining the energy to the initially calibrated value [Fig. 10 ▸(*B*) versus Fig. 10 ▸(*C*)]. Our results show that the distribution of misorientations complies with a folded-Gaussian distribution and is bound within three times the s.d. This suggests that the uncertainty in the detector orientation is in line with the uncertainty of the diffraction image in any position. Therefore, the beam direction can be assumed to be stable, and the moving-beam configuration does not add any significant aberration affecting the accuracy of the diffraction instrument. This paves the way for a more common implementation of the moving-beam diffraction geometry in new instruments.

### 3.2. Nearest-neighbor moving-beam diffraction-geometry calibration accuracy

At DIAD, the data collected by the area detector are reduced to one-dimensional intensity profiles on the basis of the diffraction geometry calibrated for the NIST standard sample in a flat-plate hollowed holder. A set of reference observations is collected with the diffraction beam stepping across the sample over a two-dimensional grid within the imaging FOV. The diffraction geometry of each observed diffractogram is independently calibrated. Data collected in routine experiments are then reduced utilizing the diffraction geometry of the nearest KB position from the reference grid. Hence, the KB position error propagates in the uncertainty of the geometry calibration.

We assess the nearest-neighbor calibration uncertainty (Table 5 ▸) by the error of the estimated interplanar distance for a diffraction ring crossing the middle of the detector panel relative to the average of those estimated with the reference self-calibrated diffraction geometry. We use the NIST SRM 660b LaB_6_ standard interplanar distance for the 210 reflection, which is *ca* 1.85903 Å. Given the error is expected to increase with the distance between beam positions of the test and nearest-neighbor reference observation, test observations are collected on a grid shifted by a half-step compared with the reference one. Hence, we measure the uncertainty associated with the largest expected geometry error.

**Figure 11:**
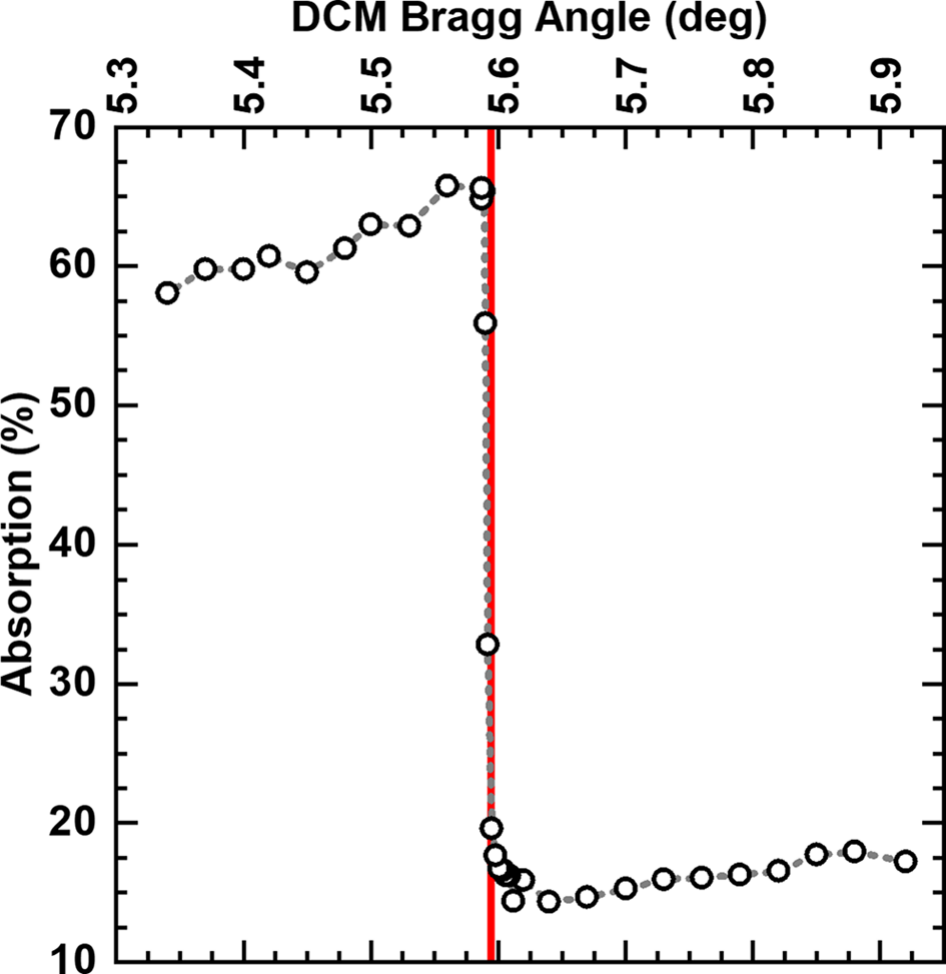
Energy calibration of the diffraction beam via observation of a standard-material absorption edge. Intensity measurements were acquired using the imaging camera with a fixed KB mirror position. The large active area of the imaging scintillator enabled full beam capture, compensating for minor beam displacements caused by changes in the DCM configuration. Measurements were taken with and without the Mo foil in the beam path, allowing normalization of the transmitted intensity to account for flux variations across different Bragg angles. These variations arise from the energy-dependent efficiency of the DCM, KB mirrors and the source emission profile.

We set the diffraction beam to a known energy to remove any source of uncertainty besides the diffraction geometry. We record the X-ray flux as a function of the DCM Bragg angle with the beam directly incident to the imaging camera and transmitted through a Mo foil. While the flux difference allows us to observe the Mo absorption energy (Fig. 11 ▸), we tune the energy of the beam for the following experiment to ∼20 keV by recovering the Bragg angle associated with the absorption edge. In addition to providing an independent energy calibration irrespective of the diffraction detector position, the close match between absorption-optimized Bragg angle and diffraction-estimated beam energy supports the reliability of the energy calibration performed with the conventional transmission detector geometry. Notably, this experiment also demonstrates the possibility of collecting space-resolved X-ray absorption near-edge structure information at DIAD.

**Figure 12:**
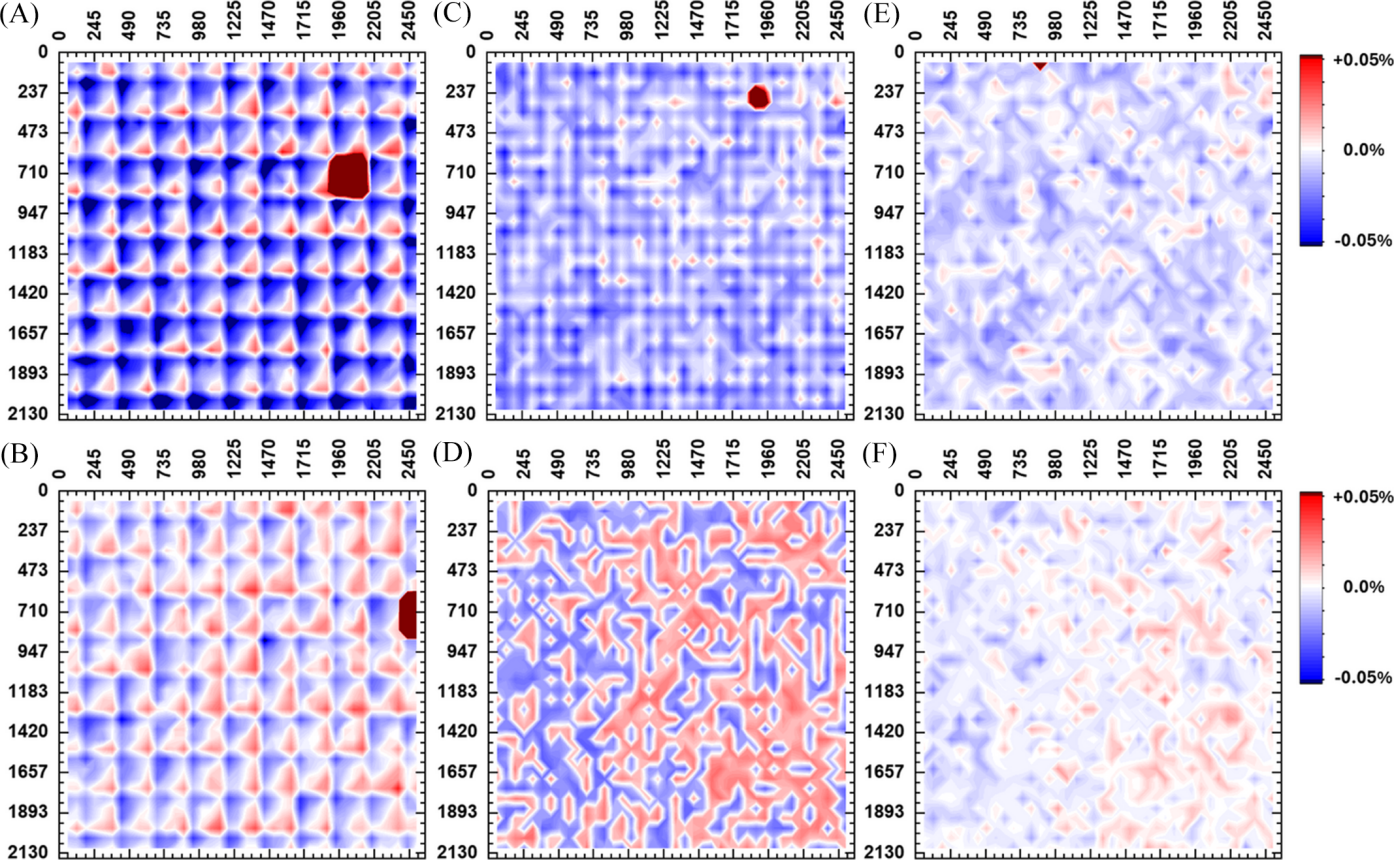
Nearest-neighbor moving-beam geometry calibration error. Interplanar-distance error estimated with the nearest-neighbor calibration relative to the average of those estimated from the reference self-calibrated diffraction geometry for flat-plate NIST SRM 660b LaB_6_, mapped on the imaging FOV (2580 × 2180 pixels). The test diffraction data are reduced using the geometry calibrated for the nearest-neighbor standard diffraction from regular Cartesian grids of (*A*), (*B*) 40 × 36, (*C*), (*D*) 20 × 18 and (*E*), (*F*) 10 × 9 beam offsets across the FOV. The diffraction beam moves across the imaging FOV on a Cartesian grid with a half-step offset compared with the denser calibration grid. The study is repeated with the detector positioned [(*A*), (*C*) and (*E*)] with conventional transmission geometry (*i.e.*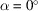, 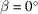, 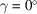 and 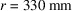) and at a standard experiment configuration [(*B*), (*D*) and (*F*)] shifted inboard without any tilt to allow room for the imaging camera to be in view of the sample (*i.e.* detector panel orthogonal to the incident-beam direction, and the pixel at the center of the detector panel at 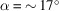, 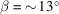, 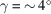 and 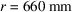). The independent self-calibrated diffraction geometry yields an s.d. of 0.008 (1)% and ∼0.006 (5)% for the two detector configurations (Table 5 ▸), respectively.

Fig. 12 ▸ shows the relative interplanar-distance error estimated across the imaging FOV for different density reference calibration grids and the two detector positions, conventional transmission and tilted relative to the incoming beam. The error is directly dependent on the distance from the reference calibration beam positions. The grid of the geometry calibration is clearly visible for the lower two density grids. As expected from Fig. 4 ▸, the conventional transmission detector geometry is characterized by larger errors compared with the case where the detector is tilted relative to the incoming beam. Notably, when the reference grid has a step size comparable to the beam spot size of 25 µm, the interplanar distance does not show any evident correlation with the KB position.

The absolute error values remain below 0.01%, which is a minimum *d*-spacing change of ∼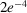 Å. This is two orders of magnitude higher than the required strain accuracy reported by Withers (2004*a* ▸,*b* ▸). Indeed, it is the same accuracy as we observed for self-calibrated datasets. Hence, a calibration grid of 40 × 36 diffraction spots evenly distributed across the imaging FOV ensures that no aberration affecting the data reduction is introduced by the nearest-neighbor geometry calibration method. To compare this error with strain-gauge resolution we need to consider the size of the strain gauge. With a limiting strain-gauge resolution of 1 µm m^−1^ and a typical size of 10 mm, the actual minimum displacement resolution is ∼100 Å. Then, the same strain resolution is achieved for a strain gauge over a 1 mm-length sample area, which is about the imaging FOV, and diffraction from a powder sample of 2 nm average size crystals. This allows DIAD to measure mechanical strain information with comparable resolution at local and macroscopic scales.

## 4. Conclusions

Here we discuss the moving-beam diffraction geometry implemented at the DIAD beamline at Diamond Light Source. Although scattering data are collected in transmission mode, the diffraction geometry is adjusted during experiments by changing the location and possibly the orientation of the incident beam. A rigid slit aperture and a focusing mirror mounted on a motion stage allow moving the center of scattering at the sample across the imaging FOV, effectively scanning the diffraction probe over the region imaged by tomography. This enables the observation of space- and time-correlated structure information avoiding perturbation of the sample, which is kept stable. However, the dynamic change of the incident-beam configuration requires calibration of the relative detector position and orientation as a function of any possible center of scattering. We achieve this via independent calibration of a dense two-dimensional grid of diffraction datasets collected from a flat-plate NIST standard calibrant, *e.g.* LaB_6_ or CeO_2_. Our measurements confirm that diffraction is most sensitive to the moving geometry for the conventional transmission detector geometry. Notably, the error induced by the moving beam is comparable to the effect of shifting the sample by an equivalent distance. Hence, the alternative option of moving the sample to scan the diffraction probe across it has no accuracy advantage compared with the moving-beam geometry, and it will be affected by slower motions and possible perturbations of the sample macrostructure (*e.g.* when including a liquid bath environment). The observational data confirm that the motion of the KB mirror coupled with a fixed-aperture slit results in a rigid translation of the beam probe, without affecting the angle of the incident-beam path to the sample. Here, we discuss the accuracy and precision of the beam stability and calibration process. We assess the reliability of the nearest-neighbor calibration method, where the data collected at any incident-beam position are reduced assuming the detector position and orientation of the closest self-calibrated standard diffraction data. Our measurements demonstrate that a nearest-neighbor calibration can achieve the same accuracy as a self-calibrated geometry when the distance between calibrated and probed sample regions is smaller than or equal to the beam spot size. We conclude that the nearest-neighbor calibration method yields an error comparable to the independent self-calibration. Notably, the error remains below 0.01%, which is a minimum *d*-spacing change of ∼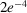 Å for a peak at *Q* of about 2 Å^−1^. This proves that the beamline achieves a suitable accuracy for measurement of structure distortion and peak shift of samples subject to change of phase and environmental conditions such as temperature and stress. Future work will further investigate the diffraction resolution for study of microstrain and phase decomposition, providing a characterization of the instrument broadening as a function of the diffraction detector position and beam energy.

## Figures and Tables

**Table 1:** Reflection sets for final position calibration and calibrated photon energy of the incident beam

SRM	Incident-beam wavelength (Å)	Reflections	Representative peak FWHM (Å^−1^)
660b LaB_6_	0.5008 (16)	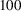 , 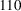 , 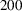 , 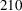 , 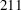 , 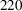 , 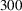 , 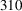 , 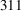 , 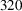 , 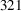 , 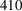 , 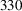 , 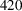 , 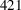	0.007
674b CeO_2_	0.41456 (11)	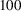 , 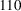 , 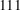 , 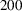 , 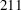 , 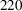 , 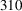 , 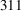 , 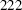 , 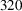 , 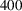	0.009

**Table 2:** Azimuthal integration parameters based on detector position are used to provide a set of one-dimensional diffraction patterns as a function of detector angle

Detector position as *r* (mm), and 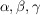 (°)	SRM	Number of azimuthal bins	Radial-range start and stop (Å^−1^)	Number of radial bins
330, 0, 0, 0	660b LaB_6_	360	1.094, 5.950	4200
300, 27, 15, 0	660b LaB_6_	120	1.094, 5.950	4200
330, 0, 0, 0	674b CeO_2_	360	1.80, 5.40	2700
300, 27, 15, 0	674b CeO_2_	120	1.80, 5.40	2700

**Table 3:** Reflections used for assessment of calibration accuracy Multiplicities have been added where multiple reflections overlap.

Detector position as *r* (mm), and 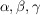 (°)	SRM	Reflection family {*hkl*}	Multiplicity	Interplanar distance (Å^−1^)
330, 0, 0, 0	660b LaB_6_	210	24	1.8593
		211	24	1.6973
300, 27, 15, 0	660b LaB_6_	210	–	–
		211	–	–
		310	24	1.3147
330, 0, 0, 0	674b CeO_2_	311	24	1.6310
		333/511	32	1.0411
300, 27, 15, 0	674b CeO_2_	311	–	–
		333/511	–	–
		642	48	0.7229
		733/555	48	0.7043

**Table 4:** Single-crystal diffraction – structure solution with *DIALS* software

Nominal *a*, *b* and *c* (Å)	4.76080 (29)	4.76080 (29)	12.99568 (87)
Estimated *a*, *b* and *c* (Å)	4.76301 (3)	4.76301 (3)	13.00170 (13)
α, β and γ (°)	90	90	120
Fast axes	0.909604	−0.0196721	0.415011
Slow axes	0.125585	−0.939136	−0.319769
Origin	7.05702	265.668	−318.778

**Table 5:** Nearest-neighbor geometry calibration uncertainty

	Conventional transmission detector geometry: 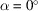 , 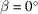 , 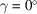 and 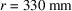		Non-conventional detector geometry:† 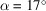 , 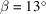 , 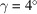 and 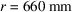	
Average distance (Å)	1.85903197		1.85895051	
	Relative error (%)		Relative error (%)	
	Average‡	S.d.	Average‡	S.d.
Self-calibrated	0.006	0.008	0.005	0.006
40 × 36	0.008	0.010	0.005	0.007
20 × 18	0.015	0.015	0.020	0.021
10 × 9	0.027	0.032	0.015	0.038

† Non-conventional: tilted relative to the incoming beam.

‡ The averages are computed over the absolute relative errors.

## Appendix A Diffraction detector-pixel coordinates

The detector plane is parametrized on the basis of an origin point situated at the top-left corner of the top-left-most pixel in the diffractogram. In the detector frame, the top-left corner position of the pixel in the *i*th column and *j*th row is

where the pixel width, *w*, and height, *h*, are both 0.172 µm for the Pilatus2M CdTe in use at DIAD.

From the detector origin, , all other pixels in the detector array are described according to the detector’s orientation  by the transformation

The detector orientation is constructed from an intrinsic composition of rotations about the three principal coordinate axes: beginning with yaw, defined as negative anticlockwise around *y*, followed by a positive anticlockwise pitch rotation about *x*′, and finally a roll negative anticlockwise around *z*′′. Considering the canonical form of the right-handed rotation matrices about the principal axes by an angle  to be ,  and , the composition is given by

Taking the incident-beam vector direction, , as  of the laboratory system, the beam center is given by

where *D* is the distance from the scattering center that the beam travels to intercept the detector plane, *i.e*. |**d**| in Fig. 3 ▸.

## Appendix B Scattering-momentum error

From Fig. 3 ▸,  and . Hence, the scattering-momentum error  simplifies to

Using equations (5) and (6), equation (14) reads

This can be simplified with equation (3) as

and

which is the same as equation (7) in Section 2.2.

## References

[bb1] Bernstein, H. J., Förster, A., Bhowmick, A., Brewster, A. S., Brockhauser, S., Gelisio, L., Hall, D. R., Leonarski, F., Mariani, V., Santoni, G., Vonrhein, C. & Winter, G. (2020). *IUCrJ* **7**, 784–792. 10.1107/S2052252520008672 PMC7467160 32939270

[bb2] Besnard, C., Marie, A., Sasidharan, S., Deyhle, H., James, A. M., Ahmed, S. I., Reinhard, C., Harper, R. A., Shelton, R. M., Landini, G. & Korsunsky, A. M. (2024). *Chem. Biomed. Imaging* **2**, 222–232. 10.1021/cbmi.3c00122 PMC10966737 38551011

[bb3] Besnard, C., Marie, A., Sasidharan, S., Harper, R. A., Shelton, R. M., Landini, G. & Korsunsky, A. M. (2023). *Dent. J.* **11**, 98. 10.3390/dj11040098 PMC10137518 37185477

[bb4] Borchert, H. (2014). *Solar cells based on colloidal nanocrystals*, Springer series in materials science, Vol. 196, pp. 79–94. Springer.

[bb5] Bragg, W. H. & Bragg, W. L. (1913). *Proc. R. Soc. A Math. Phys. Eng. Sci.* **88**, 428–438.

[bb6] Caglioti, G., Paoletti, A. & Ricci, F. P. (1958). *Nucl. Instrum.* **3**, 223–228.

[bb7] Cernik, R. J. & Bushnell-Wye, G. (1991). *Mater. Sci. Forum* **79–82**, 455–462.

[bb8] Cheary, R. W., Coelho, A. A. & Cline, J. P. (2004). *J. Res. Natl Inst. Stand. Technol.* **109**, 1–25. 10.6028/jres.109.002 PMC4849620 27366594

[bb9] Coelho, A. A. (2018). *J. Appl. Cryst.* **51**, 210–218.

[bb10] Cullity, B. D. (1956). *Elements of X-ray diffraction*, 2nd ed. Addison-Wesley Publishing Company.

[bb11] Drakopoulos, M., Connolley, T., Reinhard, C., Atwood, R., Magdysyuk, O., Vo, N., Hart, M., Connor, L., Humphreys, B., Howell, G., Davies, S., Hill, T., Wilkin, G., Pedersen, U., Foster, A., De Maio, N., Basham, M., Yuan, F. & Wanelik, K. (2015). *J. Synchrotron Rad.* **22**, 828–838. 10.1107/S1600577515003513 PMC4416690 25931103

[bb12] Ewald, P. P. (1921). *Annal. Phys.* **369**, 253–287.

[bb13] Filik, J., Ashton, A. W., Chang, P. C. Y., Chater, P. A., Day, S. J., Drakopoulos, M., Gerring, M. W., Hart, M. L., Magdysyuk, O. V., Michalik, S., Smith, A., Tang, C. C., Terrill, N. J., Wharmby, M. T. & Wilhelm, H. (2017). *J. Appl. Cryst.* **50**, 959–966. 10.1107/S1600576717004708 PMC5458597 28656043

[bb14] Guinier, A. (1956). *X-ray diffraction*. Paris: Dunod.

[bb15] Hart, M. L., Drakopoulos, M., Reinhard, C. & Connolley, T. (2013). *J. Appl. Cryst.* **46**, 1249–1260. 10.1107/S0021889813022437 PMC3778320 24068840

[bb16] Haslwanter, T. (1995). *Vision Res.* **35**, 1727–1739. 10.1016/0042-6989(94)00257-m 7660581

[bb17] He, B. B. (2009). *Two-dimensional X-ray diffraction.* Wiley.

[bb18] Horn, C., Ginell, K. M., Von Dreele, R. B., Yakovenko, A. A. & Toby, B. H. (2019). *J. Synchrotron Rad.* **26**, 1924–1928. 10.1107/S1600577519013328 PMC6853381 31721735

[bb19] Kieffer, J., Valls, V., Blanc, N. & Hennig, C. (2020). *J. Synchrotron Rad.* **27**, 558–566. 10.1107/S1600577520000776 PMC7842211 32153298

[bb20] King, A., Guignot, N., Zerbino, P., Boulard, E., Desjardins, K., Bordessoule, M., Leclerq, N., Le, S., Renaud, G., Cerato, M., Bornert, M., Lenoir, N., Delzon, S., Perrillat, J.-P., Legodec, Y. & Itié, J.-P. (2016). *Rev. Sci. Instrum.* **87**, 093704. 10.1063/1.4961365 27782575

[bb21] Kriegner, D., Matěj, Z., Kužel, R. & Holý, V. (2015). *J. Appl. Cryst.* **48**, 613–618. 10.1107/S1600576715003465 PMC4379442 25844084

[bb22] Lefmann, K. & Nielsen, K. (1999). *Neutron News* **10**(3), 20–23.

[bb23] Le Houx, J., Ruiz, S., McKay Fletcher, D., Ahmed, S. & Roose, T. (2023). *Transp. Porous Med.* **150**, 71–88. 10.1007/s11242-023-01993-7 PMC10468943 37663951

[bb24] Noell, P. J., Schindelholz, E. J. & Melia, M. A. (2020). *npj Mater. Degrad.* **4**, 32.

[bb25] Novelia, A. & O’Reilly, O. M. (2015). *Regul. Chaot. Dyn.* **20**, 729–738.

[bb26] Odstrcil, M., Lebugle, M., Lachat, T., Raabe, J. & Holler, M. (2019). *J. Synchrotron Rad.* **26**, 504–509. 10.1107/S160057751801785X PMC6412177 30855261

[bb27] Perl, J., Shin, J., Schümann, J., Faddegon, B. & Paganetti, H. (2012). *Med. Phys.* **39**, 6818–6837. 10.1118/1.4758060 PMC3493036 23127075

[bb28] Reed, J. L. A., James, A., Carey, T., Fitzgerald, N., Kellet, S., Nearchou, A., Farrelly, A. L., Fell, H. A. H., Allan, P. K. & Hriljac, J. A. (2024). *Chem. Sci.* **15**, 13699–13711. 10.1039/d4sc02664k PMC11352454 39211493

[bb29] Reinhard, C., Drakopoulos, M., Ahmed, S. I., Deyhle, H., James, A., Charlesworth, C. M., Burt, M., Sutter, J., Alexander, S., Garland, P., Yates, T., Marshall, R., Kemp, B., Warrick, E., Pueyos, A., Bradnick, B., Nagni, M., Winter, A. D., Filik, J., Basham, M., Wadeson, N., King, O. N. F., Aslani, N. & Dent, A. J. (2021). *J. Synchrotron Rad.* **28**, 1985–1995. 10.1107/S1600577521009875 PMC8570216 34738954

[bb30] Reinhard, C., Drakopoulos, M., Charlesworth, C. M., James, A., Patel, H., Tutthill, P., Crivelli, D., Deyhle, H. & Ahmed, S. I. (2022). *J. Synchrotron Rad.* **29**, 1004–1013. 10.1107/S1600577522006300 PMC9255586 35787567

[bb31] Toby, B. H. & Von Dreele, R. B. (2013). *J. Appl. Cryst.* **46**, 544–549.

[bb32] Vashishtha, H., Jamshidi, P., Vrettou, A., Kareer, A., Goode, M., Deyhle, H., James, A., Ahmad, S., Reinhard, C., Attallah, M. M. & Collins, D. M. (2024). *Mater. Charact.* **213**, 114016.

[bb33] Warren, B. E. (1990). *X-ray diffraction*. Dover Publications.

[bb34] Waterman, D. G., Winter, G., Gildea, R. J., Parkhurst, J. M., Brewster, A. S., Sauter, N. K. & Evans, G. (2016). *Acta Cryst.* D**72**, 558–575. 10.1107/S2059798316002187 PMC4822564 27050135

[bb35] Willendrup, P., Farhi, E., Knudsen, E., Filges, U. & Lefmann, K. (2014). *J. Neutron Res.* **17**, 35–43.

[bb36] Willendrup, P., Farhi, E. & Lefmann, K. (2004). *Physica B* **350**, E735–E737.

[bb37] Willendrup, P. K. & Lefmann, K. (2020). *J. Neutron Res.* **22**, 1–16.

[bb38] Willendrup, P. K. & Lefmann, K. (2021). *J. Neutron Res.* **23**, 7–27.

[bb39] Withers, P. J. (2004*a*). *J. Appl. Cryst.* **37**, 596–606.

[bb40] Withers, P. J. (2004*b*). *J. Appl. Cryst.* **37**, 607–612.

[bb41] Withers, P. J., Daymond, M. R. & Johnson, M. W. (2001). *J. Appl. Cryst.* **34**, 737–743.

[bb42] Wright, J. P., Giacobbe, C. & Lawrence Bright, E. (2022). *Crystals* **12**, 255.

[bb43] Yang, L., Liu, J., Chodankar, S., Antonelli, S. & DiFabio, J. (2022). *J. Synchrotron Rad.* **29**, 540–548. 10.1107/S1600577521013266 PMC8900859 35254319

